# Telomeric Repeat-Binding Factor Homologs in *Entamoeba histolytica*: New Clues for Telomeric Research

**DOI:** 10.3389/fcimb.2018.00341

**Published:** 2018-10-02

**Authors:** Francisco Javier Rendón-Gandarilla, Víctor Álvarez-Hernández, Elizabeth J. Castañeda-Ortiz, Helios Cárdenas-Hernández, Rosa Elena Cárdenas-Guerra, Jesús Valdés, Abigail Betanzos, Bibiana Chávez-Munguía, Anel Lagunes-Guillen, Esther Orozco, Lilia López-Canovas, Elisa Azuara-Liceaga

**Affiliations:** ^1^Posgrado en Ciencias Genómicas, Universidad Autónoma de la Ciudad de Mexico, Mexico City, Mexico; ^2^Departamento de Bioquímica, Centro de Investigación y de Estudios Avanzados del Instituto Politécnico Nacional, Mexico City, Mexico; ^3^Consejo Nacional de Ciencia y Tecnología, Mexico City, Mexico; ^4^Departamento de Infectómica y Patogénesis Molecular, Centro de Investigación y de Estudios Avanzados del Instituto Politécnico Nacional, Mexico City, Mexico

**Keywords:** TRF, Myb-like DNA binding domain, lamin B1, H4K20, chromosome, DNA sequence

## Abstract

Telomeric Repeat Binding Factors (TRFs) are architectural nuclear proteins with critical roles in telomere-length regulation, chromosome end protection and, fusion prevention, DNA damage detection, and senescence regulation. *Entamoeba histolytica*, the parasite responsible of human amoebiasis, harbors three homologs of human TRFs, based on sequence similarities to their Myb DNA binding domain. These proteins were dubbed EhTRF-like I, II and III. In this work, we revealed that EhTRF-like I and II share similarity with human TRF1, while EhTRF-like III shares similarity with human TRF2 by *in silico* approach. The analysis of *ehtrf-like* genes showed they are expressed differentially under basal culture conditions. We also studied the cellular localization of EhTRF-like I and III proteins using subcellular fractionation and western blot assays. EhTRF-like I and III proteins were enriched in the nuclear fraction, but they were also present in the cytoplasm. Indirect immunofluorescence showed that these proteins were located at the nuclear periphery co-localizing with Lamin B1 and trimethylated H4K20, which is a characteristic mark of heterochromatic regions and telomeres. We found by transmission electron microscopy that EhTRF-like III was located in regions of more condensed chromatin. Finally, EMSA assays showed that EhTRF-like III forms specific DNA-protein complexes with telomeric related sequences. Our data suggested that EhTRF-like proteins play a role in the maintenance of the chromosome ends in this parasite.

## Introduction

Telomeres are specialized protein-DNA complexes localized at the end of eukaryotic chromosomes (Blackburn and Gall, 1978; Meyne et al., 1989; Giraud-Panis et al., 2013). Telomeric DNA consists of tandem arrays of G + T-rich repetitive sequences ending in a single-stranded G-rich overhang which is added by the ribonucleoprotein enzyme telomerase (O'Sullivan and Karlseder, 2010; Giraud-Panis et al., 2013). The length of these arrays varies among species, ranking from few hundred base pairs disposed in irregular tandem repeats in *Saccharomyces cerevisiae* (Lue, 2010), to thousands of base pairs of a TTAGGG repeat in vertebrate telomeres (Moyzis et al., 1988; Giraud-Panis et al., 2013). Proteins that bind to telomeric DNA play critical roles in telomere length regulation and chromosomal end protection in eukaryotic organisms. They conform a machinery known as Telosome or Shelterin complex (Palm and de Lange, 2008). These protein complexes include members or functional homologs of the TTAGGG Repeat Binding Factors, Telomeric Repeat Binding Factors (TRF) and telobox family members (Chong et al., 1995; Broccoli et al., 1997b). TRF proteins are architectural nuclear proteins involved in diverse roles, such as telomere length regulation, chromosome end protection, prevention of chromosomes fusion, sense of DNA damage, and regulation of senescence (de Lange, 2005; Palm and de Lange, 2008). These proteins are conserved from lower eukaryotes, to plants and mammals (Horvath, 2000–2013). The genome *of H. sapiens* contains two genes coding for TRF proteins: TRF1 and TRF2, these proteins bind as homodimers to the double-stranded DNA telomeric sequence (Chong et al., 1995; Broccoli et al., 1997b). TRF1 controls the length of telomeric repeats, whereas TRF2 is involved in the assembly of the terminal t-loop, negative telomere length regulation and chromosomal end protection (Palm and de Lange, 2008). Proteins of the TRF family have similar architectures, defined by the presence of two domains: (i) the conserved single MYB-type helix-turn-helix (HTH) DNA-binding domain (MYB DBD; 55 amino acids) located in their C-terminal; this domain contains three evenly spaced tryptophan residues and in the third α-helix presents a telobox motif (VDLKDKWRT, consensus VxxKDxxR) (Bilaud et al., 1996). (ii) The TRF-homology domain (TRFH; 200 amino acids) situated in the N-terminal, which is unique for members of this family (Broccoli et al., 1997b); and its function is related to homodimerization and protein-protein interactions with other telomeric proteins (Fairall et al., 2001). Additionally, TRF1 and TRF2 proteins diverge in their N-terminal domain, which is rich in acidic or basic residues, respectively (Broccoli et al., 1997a; Palm and de Lange, 2008). Gene encoding homologs to TRF1 and TRF2 have been found in the genomes of *Trypanosoma brucei, Trypanosoma cruzi* and *Leishmania. major* based on similarities to the C-terminal MYB DBD (Li et al., 2005; da Silva et al., 2010). Besides TRF1 and TRF2, telomeric DNA also requires the binding of other specific proteins, such as Rap1 (replication protein A 1), POT1 (protection of telomeres 1), TIN2 (TRF1-interacting nuclear factor 2) and TPP which together conform the Shelterin complex in *H. sapiens* (Palm and de Lange, 2008). In Trypanosomes besides the TRF2, a homolog of Rpa-1 has also been identified, suggesting that the telomeric function is conserved and that the telomeric machinery evolved early in eukaryotes (Lira et al., 2007).

In *Entamoeba histolytica*, the causative protozoan of human amoebiasis, the MYB DBD is the most abundant domain related to transcriptional regulation (Clark et al., 2007). MYB DBD-containing proteins in this parasite are clustered into three monophyletic groups. Families I and III are related to transcriptional factors and were dubbed as EhMybR2R3 and EhMybSHAQKYF, respectively (Meneses et al., 2010). Family II includes single-repeat proteins related to human telomeric binding proteins (Meneses et al., 2010). In *E. histolytica* the identification of telomeric signatures has been a challenging task. Although the first draft of *E. histolytica* genome was published in 2005, it has not been possible to identify sequences that referenced the terminal ends of the chromosomes neither canonical telomeric sequences nor orthologs of telomerase genes have been identified (Loftus et al., 2005; Clark et al., 2007; Lorenzi et al., 2010). However, 10% of the *E. histolytica* genome corresponds to tRNA genes which are associated with short tandem repeats (Loftus et al., 2005). There are 25 different types of long tandem arrays that contain between 1 and 5 tRNA types per repeat unit and STRs which resembles microsatellites (Clark et al., 2006; Tawari et al., 2008). It has been proposed that these arrays could localize at the chromosome ends, acting as telomeric regions that fulfill a structural role in the genome (Clark et al., 2006; Tawari et al., 2008). In addition, *E. histolytica* chromosomes do not completely condense and there is a considerable variation in their chromosome size, maybe due to expansion and contraction of telomeric repeats, as in other protists (Patarapotikul and Langsley, 1988; Melville et al., 1999; Willhoeft and Tannich, 2000). Until now, no telomeric sequences or protein complexes implicated in telomere function have been described in this parasite. The study of TRF homologs will help to gain insight into telomere biology of *E. histolytica*. Thus, in this work, we identified and characterized the TRF-like proteins of *E. histolytica* as homologous to human TRF1 and TRF2. We observed their nuclear localization in condensed chromatin regions, their co-localization with Lamin B1 and trimethylated H4K20, and their binding capacity to form DNA-protein complexes with telomeric related sequences. Our results suggest that the TRF-like proteins of *E. histolytica* play similar function as in their human counterparts. However, further experiments are still needed to address their role in the maintenance of telomeres in this parasite.

## Materials and methods

### *E. histolytica* cultures

*E. histolytica* trophozoites (strain HM1:IMSS) were axenically cultured at 37°C in TYI-S-33 medium, supplemented with 15% bovine serum (Microlab), 1% Diamond vitamin mixture (Sigma-Aldrich), 100 μg ml^−1^ streptomycin sulfate and 100 U ml^−1^ penicillin (Diamond et al., 1978). Trophozoites were harvested during logarithmic growth phase.

### *In silico* analysis of the EhTRF-like proteins of *E. histolytica*

Amino acid sequences of proteins coded by genes from *locus* EHI_001090, EHI_148140 and EHI_001110, EHI_009820 and EHI_074810 were obtained from AmoebaDB database (http://amoebadb.org/amoeba/). Such sequences were used as a bait to perform queries using the DELTA-BLAST (Domain Enhanced Lookup Time Accelerated BLAST) algorithm to identify orthologs in other members of Entamoeba or other eukaryotes (https://blast.ncbi.nlm.nih.gov/Blast.cgi). The Percent of Identity (PID) was calculated using the amino acids sequences from *E. histolytica* proteins coded by the above-mentioned genes, TRF1, TRF2, and proteins from organisms corresponding with the best hits, taking gaps into account using the following equation: PID = (identical positions/length of the alignment) × 100. The predicted secondary structure of complete TRF1 and EhTRF-like I, II and III (corresponding to AmoebaDB ID: EHI_001090, EHI_001110, and EHI_148140, respectively) proteins was determined using PSIPRED (http://bioinf.cs.ucl.ac.uk/psipred/) tool and aligned by ClustalW2 software (https://www.ebi.ac.uk/Tools/msa/clustalw2/). Molecular weight, pI and post-translational modifications were analyzed using the ExPaSy: Compute pI/Mw (https://web.expasy.org/compute_pi/), ProtPi (https://www.protpi.ch/) and Mod Pred tools (http://www.modpred.org/). Nuclear localization signals (NLS) were located through SeqNLS (http://mleg.cse.sc.edu/seqNLS/) and cNLS (http://nls-mapper.iab.keio.ac.jp/cgi-bin/NLS_Mapper_help.cgi) tools. The amino acid sequence of DBD MYB from TRF proteins was aligned using ClustalW2 and phylogenetic analysis was inferred using Neighbor-Joining method. The evolutionary distances were computed using the Poisson correction method. Evolutionary analyses were conducted using MEGA 7 (Kumar et al., 2016). Phylogenetic tree was constructed through a bootstrap of 1,000 replicates. To identify orthologous components of the Sheltering machinery in *E. histolytica* genome, the amino acid sequences of *Homo sapiens* Rap1 (Q9NYB0), TIN2 (Q9BSI4), TPP1 (Q96AP0), and POT1 (Q9NUX5) were obtained from the UniProt database (http://www.uniprot.org/) and used as a bait to make queries with Blast algorithm (http://blast.ncbi.nlm.nih.gov). The presence of conserved functional domains in the identified proteins was analyzed in the Pfam database (http://pfam.xfam.org/).

### RT-PCR assays

Total RNA from *E. histolytica* trophozoites was isolated following the Trizol® LS Reagent (Invitrogen) protocol and then semi-quantitative RT-PCR assays were performed with 100 ng of total RNA. Primers used to amplify the *ehtrf-like I, ehtrf-like II, ehtrf-like III* and *40s rps2* genes were designed using Primer-BLAST (https://www.ncbi.nlm.nih.gov/tools/primer-blast/) and manually corrected (Table S1). To validate the specificity of these primers, we amplified the specific sequence from 50 ng of genomic DNA, obtained with the Wizard Genomic purification kit (Promega). PCR products were separated by gel electrophoresis in 2% agarose gels, stained with RedGel™ Nucleic AcidGel Stain 10,000X (BIOTUM) and visualized in a standard UV transilluminator. Relative quantification by qRT-PCR was performed using the QuantiFast SYBR Green RT-PCR kit (Qiagen) and 50 ng of the total RNA, according to the manufacturer's instructions. Relative changes in gene expression were calculated using *40s rps2* as an internal gene calculated by ΔΔCT method. Absolute quantitation was performed using a 10-fold serially diluted standard curve of each *ehtrf-like I, II, III* and *40s rps2* genes in parallel with qRT-PCR of RNA from trophozoites grown in basal conditions. Reaction volumes were set with 25 μl QuantiFast SYBR Green RT-PCR kit (Qiagen) and qRT-PCR was performed using 50 ng of the total RNA and 1 μM each primer, according to the manufacturer's instructions. Initial thermal cycling conditions were 1 cycle of 50°C for 10 mins for reverse transcription, followed by 1 cycle of 95°C for 5 min and 40 cycles of denaturation at 95°C for 10 s and annealing/extension temperature of 55°C for 30 s. Plotting Ct values vs. copy number of the different genes in a standard curve allowed to approximate copies *ehtrf-like* genes from Ct values. The data shown was displayed as mean with standard error in triplicate and repeated in independent experiments by duplicate. GraphPad Prism 6.0e software was used for student *t*-test by two-tailed analyses.

### Production of polyclonal antibodies against EhTRF-like i and EhTRF-like III

The complete amino acid sequences from EhTRF-like I and EhTRF-like III proteins were aligned using the ClustalW2 tool to identify unique regions of these proteins. Subsequently, to determine hydrophobic regions, EhTRF-like I and III amino acid sequences were analyzed using the Hopp-Woods program (Hopp and Woods, 1981). Finally, a prediction of B epitopes was made using the ABCpred server (http://crdd.osdd.net/raghava/abcpred/). Differential peptides, CTLPSVGNALIPPS and CNKQKVQPQVSQPH for EhTRF-like I and III, respectively, were synthesized, conjugated to Keyhole Limpet Hemocyanin (KLH) (GL Biochem, Shanghai) and used to immunize New Zealand male rabbits. Before immunization, the pre-immune serum (PS) was obtained and then rabbits were subcutaneously inoculated with 400 μg of each peptide diluted in TiterMax® Gold (Sigma-Aldrich). Four booster injections (500 μg each) at 15-days intervals, were given. After 1 week of the last immunization, rabbits were bled, and polyclonal antisera were obtained and tested by western blot assays against total extracts of *E. histolytica* trophozoites.

### Subcellular fractionation

*E. histolytica* trophozoites were recovered by centrifugation and lysed as follows to obtain subcellular fractions. Soluble nuclear extracts (Ns) were prepared as described by Schreiber et al. (1989). Briefly, 1 × 10^6^ trophozoites were resuspended by gentle pipetting in 400 μl of cold buffer A (10 mM HEPES pH 7.9, 10 mM KCl, 0.1 mM EDTA, 0.1 mM EGTA, 1 mM DTT, 0.5 mM PMSF). Trophozoites were allowed to swell on ice for 15 min, after which 25 μl of 10% NP-40 solution (Sigma-Aldrich) was added and vigorously vortexed for 30 s. Homogenate was centrifuged for 10 min at 14,000 rpm, to separate the supernatant containing cytoplasmic extracts (C), and the pellet containing nuclei. Pellet was resuspended in 1 ml of buffer A and layered on 1 ml of buffer A containing 0.34 M sucrose (Sigma-Aldrich), then mixed and centrifuged for 10 min at 14,000 rpm. The nuclear pellet was resuspended in 150 μl ice-cold buffer C (20 mM HEPES pH 7.9; 0.4 M NaCl; 1 mM EDTA; 1 mM EGTA; 1 mM DTT; 1 mM PMSF) and vigorously rocked at 4°C for 15 min. Nuclear soluble extracts (Ns) were obtained after centrifugation for 10 min at 13,000 rpm at 4°C and frozen in aliquots at −70°C until used. The remaining pellet was lysed in RIPA buffer (50 mM Tris-Cl, pH 8, 150 mM NaCl, 2 mM EDTA, 1% Triton X-100, 0.1% SDS) and sonicated with three pulses of 30 s (30% power) and 2 min refractory period each on ice, to disrupt the chromatin. Later, samples were centrifuged at 14,000 rpm at 4°C for 10 min and supernatants were labeled as nuclear insoluble fractions (Ni). Total extracts (T) were obtained by freeze-thawing trophozoites in the presence of 100 mM Tris-HCl pH 7.9, 100 mM p-hydroxymercuribenzoate (PHMB) (Sigma-Aldrich), Complete™ protease inhibitor cocktail (Sigma-Aldrich), 10 μM E64 (Sigma-Aldrich) and 100 mM PMSF (Sigma-Aldrich). Protein concentration was determined by Bradford method (Bradford, 1976) using the Bio-Rad protein assay or the DC Protein Assay (BIO-RAD).

### SDS-PAGE gels and western blot assays

Protein extracts obtained as previously indicated (T, Ns, Ni or C), were separated by sodium dodecyl sulfate polyacrylamide gel, electrophoresed by (SDS-PAGE). Briefly, 40 μg of each extract was loaded in a 12% SDS-PAGE and transferred to 0.45 μm nitrocellulose membranes (BIO-RAD). For western blot (WB) assays, membranes were stained with Ponceau S Red staining solution (Sigma-Aldrich) and incubated 1 h with 5% non-fat milk. Then were incubated overnight (ON) with α-TRF-like I (1:2,500), α-TRF-like III (1:1,000), α-EhKMT4 (1:1,000) (Borbolla-Vázquez et al., 2015); α-H3K27me3 (1:1,000, Cell Signaling Technology), α-H4K20me3 (1:1,000, Abcam), α-Lamin B1 (1:1,000, Abcam), α-actin (1:1,000, Sigma-Aldrich) or α-Myc (1:1,000, Cell Signaling Technology) antibodies and then, incubated for 2 h with the corresponding horseradish peroxidase (HRP) labeled secondary antibodies (1:8,000, Santa Cruz Biotechnology). Protein bands were detected and visualized by chemiluminescence on X-ray films. All experiments were repeated at least three times.

### Subcellular localization by confocal microscopy

Trophozoites cultured on cover slides were fixed with absolute ethanol for 20 min at −20°C and washed with PBS pH 6.8. Then, coverslips were incubated with 50 mM NH_4_Cl for 30 min at 37°C and blocked with 1% bovine serum albumin for 30 min. For co-localization experiments, α-EhTRF-like I, α-Lamin B1, α-EhTRF-like III and α-H4K20me3 antibodies were labeled with Alexa Flour 647, 555, 488 and 488, respectively, using Molecular Probes® Antibody Labeling kit (ThermoFisher Scientific) and following the manufacturer's instructions. In other experiments, samples were incubated ON at 4°C with α-TRF-like III (1:200) and α-Myc (1:100, Cell Signaling) antibodies. Cells were washed and then, incubated with fluorescein labeled secondary antibody (1:200, Jackson Immuno Research). Slides were mounted with Vectashield containing DAPI (Vector Lab), visualized through a Nikon inverted microscope attached to a laser Confocal scanning system (Leica TCS SP2) and analyzed by Confocal Assistant software image.

### Construction of *pehtrf-like Iox, pehtrf-like IIIox*, and *pCold-ehtrf-like I* plasmids

The *ehtrf-like I* (1,210 pb) and *ehtrf-like III* (1,383 bp) complete open reading frames (ORF) were cloned in the *pKT-3M* (Saito-Nakano et al., 2004) plasmid to express the EhTRF-like I and III proteins tagged with Myc sequence at their N-terminal. The *ehtrf-like I* gene was amplified by PCR using the following oligonucleotides: sense 5′-TCCCCCCGGGATGAATAACCCTCAGTTGC-3′ and antisense 5′- GCGCGCCTCGAGTTATTGAGAAAGATCCAATTGTTTAAAT-3′. The *ehtrf-like III* gene was amplified using the following oligonucleotides: sense 5′-TCCCCCCCGGGATGGAGAAAAAACTAA-3′ and antisense 5′-GGGGCCTCGAGTTAAAATTATCAGAATTA-3′. Forward primers contained a *Sma*I site and the reverse primers contained a *Xho*I site (underlined). PCR was performed with 100 ng of *E. histolytica* genomic DNA, using the following conditions: 92°C for 5 min, 92°C for 1 min, 55°C for 1 min, and 70°C for 1 min (28 cycles). PCR products were digested with *Sma*I and *Xho*I and cloned into a previously digested *pKT-3M* vector. For the EhTRF-like I recombinant protein (rEhTRF-like I), the 1,210 pb entire ORF was amplified using the forward primer 5-CGCGGATCCTTATTGAGAAAGATCCAATTG-3 and reverse primer 5-GGAATTCCATATGAATAACCCTCAGTTTGC-3, which contained *Bam*HI and *Nde*I restriction sites, respectively. The 1,210 bp amplicon was digested and cloned into a previously linearized *pCold I* vector (Takara). All plasmids obtained were confirmed by sequencing using the BigDye Terminator v3.1 Cycle Sequencing Kit and the ABI Prism® 310 Genetic Analyzer (Applied Biosystems).

### Transfection of trophozoites

Trophozoites were transfected by lipofection as previously described (Olvera et al., 1997; Abhyankar et al., 2008). Briefly, 8 × 10^5^ trophozoites were placed for adhesion in 6-well plates during 20 min, and then washed with M-199 medium (Invitrogen) supplemented with 5.7 mM cysteine, 1 mM ascorbic acid and 25 mM HEPES pH 6.8. Later, 20 μg of *pKT-3M or pehtrf-like Iox* or *pehtrf-like IIIox* plasmids were added to a tube containing 20 μl of Superfect (Qiagen), incubated at room temperature (RT) for 20 to 30 min, mixed with 0.8 ml of M199 supplemented medium plus 15% bovine serum and added to each well-plate. Trophozoites were incubated for 4 h at 37°C and then harvested and added to a 125 mm cell culture tube, containing pre-warmed TYI-S-33 medium. Transfected trophozoites were grown for 48 h and selected initially with 3 μg ml^−1^ G-418 (Thermofisher Scientific) and gradually increased to 20 μg ml^−1^.

### Immunoelectron microscopy

Immunoelectron microscopy was performed as described (Segovia-Gamboa et al., 2011). Briefly, transfected trophozoites were washed twice with PBS, pH 7.4 and fixed in 4% paraformaldehyde and 0.1% glutaraldehyde in serum-free medium for 1 h at RT. Samples were embedded in LR White resin and polymerized under UV at 4°C ON. Sections were obtained and mounted on formvar-covered nickel grids. Then, they were incubated with α-EhTRF-like III (1:1,000) and α-Myc (1:1,000) antibodies, followed by incubation with 30 nm gold-conjugated particles (Ted Pella Inc.) secondary antibodies (1:60). Thin sections (60–90 nm) were observed in a transmission electron microscope (JEM 1011).

### Expression and purification of rEhtRF- like i

*Escherichia coli* Rosetta (DE3) competent cells (Novagen) were transformed with the *pCold-ehtrf-like I* construct. The expression of rEhTRF-like I was induced with 1 mM isopropyl-1-thio-β-D-galactopyranoside (IPTG) at 16°C for 18 h and analyzed by SDS-PAGE and WB assays. The WB assays were performed by using an anti-histidine monoclonal antibody (α-His) (Santa Cruz Biotechnology, Inc) (1:1,000) as the primary antibody and a peroxidase-conjugated goat anti-mouse polyclonal secondary antibody (Invitrogen-Gibco) at a dilution of 1:3,000. The recombinant protein was obtained from the soluble fraction and purified using an affinity chromatography by Ni Sepharose High Performance agarose (GE Healthcare), following the manufacturer's instructions. The rEhTRF-like I was dialyzed with 25 mM Tris–HCl pH 7.5 and 300 mM NaCl. Protein was quantified and used as described below.

### Electrophoresis mobility shift assays (EMSA)

DNA-binding activity was tested with four different DNA probes: HsTEL [TTAGGG, human telomeric sequence (Hwang et al., 2001)], EhTEL (the STR sequence TTAGTATT derived from the NK2 unit from the tRNA arrays of *E. histolytica*; Clark et al., 2006; Tawari et al., 2008), a mut-TEL (mutated telomeric sequence) and non-Rel (non-related sequence) probe (Table S2). Double-stranded oligonucleotides were end-labeled with 10 μCi [α-^32^P] dATP (Perkin Elmer) or 10 μCi [γ-^32^P] dATP, using Klenow fragment or a polynucleotide kinase (New England Biolabs) and purified with the QIAquick nucleotide removal kit (Qiagen). Nuclear soluble extracts (10 μg) from wild type or transfected trophozoites or 106.86 μM rEhTRF-like I were incubated for 20 min at 4°C with 1 ng of labeled oligonucleotides in binding buffer (12 mM HEPES pH 7.9, 60 mM KCl, 5 mM MgCl_2_, 1 mM EDTA, 4 mM Tris–HCl, 5 mM DTT, 10% glycerol, 4 mM Spermidine, 4 mM MgCl, 50 ng of poly dI·dC and protease inhibitors cocktail). Competitions were carried out in the presence of a 50, 100 and 200-fold molar excess of unlabeled specific HsTEL, EhTEL, mut-Tel and non-Rel probes. Supershifts were performed by pre-incubating nuclear extracts from transfected trophozoites with α-EhTRF-like III or PS (2–4 μg/reaction) for 30 min on ice, before adding the probe. DNA–protein complexes were resolved on a 6% non-denaturing PAGE gel by electrophoresis at 160 V for 2 h at RT in 0.5 × TBE buffer (44.5 mM Tris-borate, 44.5 mM boric acid, 1 mM EDTA). Gels were vacuum-dried and radioactive complexes were detected in a Phosphor Imager apparatus (BIO-RAD) or revealed by autoradiography using Kodak X-omat film (Sigma) exposed at −70°C.

### Ethic statement

The Institutional Animal Care and Use Committee (Cinvestav IACUC/ethics committee) reviewed and approved our protocol for the animal care and use of rabbits employed to produce antibodies (Protocol Number 0313-06, CICUAL 001). All steps were taken to ameliorate the welfare and to avoid the animals suffering. Food and water were available *ad libitum*. Animals were monitored pre- and post-inoculation. All procedures were conducted by trained personnel under the supervision of veterinarians and, all invasive clinical procedures were performed while animals were anesthetized and when it was required, animals were humanely euthanized. The ethics committee verified that our Institute fulfills the NOM-062-ZOO-1999, regarding the Technical Specifications for Production, Care and Use of Laboratory Animals given by the General Direction of Animal Health of the Minister of Agriculture of Mexico (SAGARPA-Mexico). The technical specifications approved by SAGARPA-Mexico fulfill of the international regulations/guidelines for use and care of animals used in laboratory and were verified and approved by Cinvestav IACUC/ethics committee (Verification Approval Number: BOO.02.03.02.01.908).

## Results

### *In silico* characterization of *E. histolytica* EhTRF-like proteins

In this work, we focus on the family II of the MYB DBD-containing proteins of *E. histolytica* (Meneses et al., 2010). This family comprises genes encoding hypothetical proteins with AmoebaDB accession numbers (AE): EHI_001090, EHI_001110, EHI_148140, EHI_009820, and EHI_074810, which were reported as single-repeat telomeric proteins related to human telomeric binding proteins (Meneses et al., 2010). On one hand, analysis of the amino acid sequences of the proteins coded by genes from *locus* EHI_009820 and EHI_074810 showed identities with the MYB DBD-containing proteins related to c-Myb, with narrowed identity to TRF-related proteins (Table 1). In contrast, the amino acid sequences of proteins coded by genes from *locus* EHI_001090, EHI_001110, and EHI_148140 present about 25.8 to 29% identity with TRF1 from *H. sapiens* (Table 1). Moreover, these proteins share 29.6 to 30.3% identity with TRF proteins from other organisms, such as *Pan troglodytes* or *Desmus rotundus* (Table 1). Therefore, we considered these *E. histolytica* hypothetical proteins as TRF-like proteins and from now we named them as EhTRF-like I (EHI_001090), II (EHI_001110), and III (EHI_148140). *E. histolytica* EhTRF-like proteins showed higher homology between each other than with human homologs. The sequence conservation between full length EhTRF-like proteins was 35.89 to 63.94% identity, in contrast to human TRF1 and TRF2 which had a 27.77% identity between them. Interestingly, the Myb DBD domain, which characterizes TRF proteins, showed 80.32 to 93.44% identity in EhTRF-like proteins, exhibiting a higher identity between EhTRF-like I and II (Table S3).

**Table 1:** Comparison of MYB DBD-containing proteins of *E. histolytica* with other organisms.

***Entamoeba histolytica***			***Homo sapiens***				**Other organisms**			
**Locus^∧^**	Annotation*	**Protein**	**Accession**	***e*-value**	**PID^&^**	**Protein**	**Species**	**Accession**	***E*-value**	**PID**
EHI_009820	Myb-like	c-Myb	Q708E3	7e-13	24	TFIIIB	*Camponotus floridanus*	E2ACRO	4e-13	34.7
EHI_074810	HP^#^	c-Myb	Q708E3	2e-10	29	Myb-like	*Tetrahymena thermophila*	Q23Q10	9.1e-9	35.1
EHI_148140	HP^#^	TRF1	P54274	4.8e-7	29.7	TRF1	*Pan troglodytes*	H2R473	2.2e-8	30.3
EHI_001090	HP^#^	TRF1	P54274	4.8e-7	29.7	TRF1	*Pan troglodytes*	H2R473	2.2e-8	30.3
EHI_001110	HP^#^	TRF1	P54274	9.9e-7	25.8	TRF1	*Desmodus rotundus*	K9J0W4	6.6e-10	29.6

∧ *According to AmoebaDB data base*.

* According to Uniprot.

& *Percentage of identity in global alignment*.

# *Hypothetical protein*.

**Figure 1:**
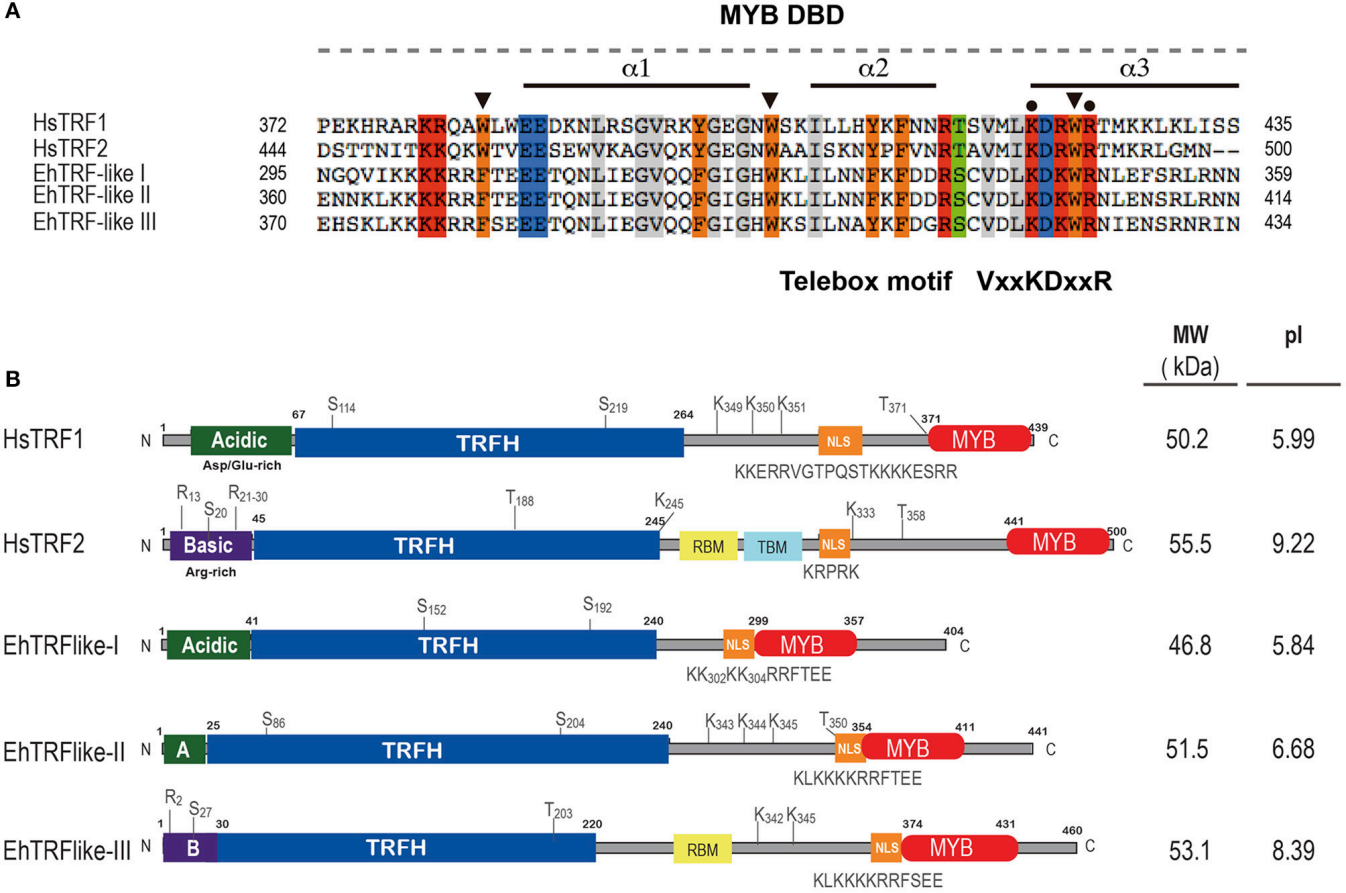
EhTRF-like proteins in *E. histolytica*. **(A)** Multiple alignment of MYB DBD from *Homo sapiens* (HsTRF2 and HsTRF1) and *E. histolytica* (EhTRF-like I, II and III). Black bars illustrate the position of the three α-helices in MYB DBD. Conserved tryptophans or aromatic residues are marked with a black arrowhead. Telebox consensus is shown, conserved amino acids are marked with black dots and identical amino acids are highlighted in colors. **(B)** Schematic representation of major domains and post-translational modifications contained in TRF1 proteins. TRFH, dimerization domain; NLS, nuclear localization signal; MYB DBD, Myb-like DNA binding domain; A, acidic N-terminus; B, basic N-terminus; MW, molecular weight; and pI, isoelectric point.

The alignment of the MYB DBD of the EhTRF-like proteins with TRF1 and TRF2 from *H. sapiens* showed that the sequences conserved the three α-helices characteristics of the MYB DBD, as well as the positions of the second and third tryptophan residues responsible of HTH conformation (Figure 1A; Figure S1). In addition, EhTRF-like I, II and III have a telebox motif (VxKDxxR) in the third α-helix, and a lysine and arginine residues involved in the DNA telomeric recognition in human TRF1 and TRF2 (Figure 1A; Figure S1).

Even though there was scarce amino acid identity among the TRFH domains from EhTRF-like proteins and human TRFs, several hydrophobic residues required for the dimer interface, such as leucines, isoleucines, and phenylalanines were conserved (Figure S1). In addition, we deduced the secondary structure of the complete amino acid sequences for EhTRF-like proteins and human TRF1 and we aligned them to have a structural comparison (Figure S2). Based on their secondary structure, we proposed the presence of a TRFH domain located in the N-terminal of EhTRF-like proteins, because we found at the same position, the nine α-helices essential for domain dimerization in human TRF1 (Figure 1B; Figure S2).

According to the ORFs size (1,215, 1,326, and 1,383 pb for *ehtrf-like I, II* and *III*, respectively), the predicted molecular weight of EhTRF-like I, II and III is 46.8, 51. 5, and 53.1 kDa, respectively, showing similarity to their human counterparts (Figure 1B). TRF1 and TRF2 have isoelectric points (pI) of 5.99 and 9.22, respectively, which are related to their amino acids content in their N-terminal end. Theoretical pI of EhTRF-like I and II were predicted as 5.84 and 6.68, respectively, similar to that of TRF1, which is an acidic protein. On the contrary, EhTRF-like III presented a predicted pI of 8.39, alike to the basic protein TRF2. Consequently, the first 50 amino acids of EhTRF-like I and II were acidic residues, similar to those found at the same region of TRF1. On the other hand, the EhTRF-like III N-terminal was composed of basic residues as in TRF2 (Figure 1B). TRF2 also contains binding sites for the Shelterin proteins, Rap1 and TIN2 (de Lange, 2005). In concordance, in EhTRF-like III we identified the presence of two α-helices with conserved hydrophobic residues, corresponding to a Rap1-binding domain (RBM domain; Figure 1B). Likewise, all EhTRF-like proteins showed a nuclear localization signal (NLS) and susceptible sites of phosphorylation and SUMOylation, present in their human counterparts (Figure 1B). Altogether, we predict that EhTRF-like proteins from *E. histolytica* have a similar architecture to TRF1 and TRF2 from *H. sapiens* and are homologs to mammalian telomeric repeat-binding factors. In summary, EhTRF-like I and II share common properties with TRF1, while EhTRF-like III does with TRF2.

### Phylogenetic analysis of TRF proteins with MYB DBD or telebox domain

**Figure 2:**
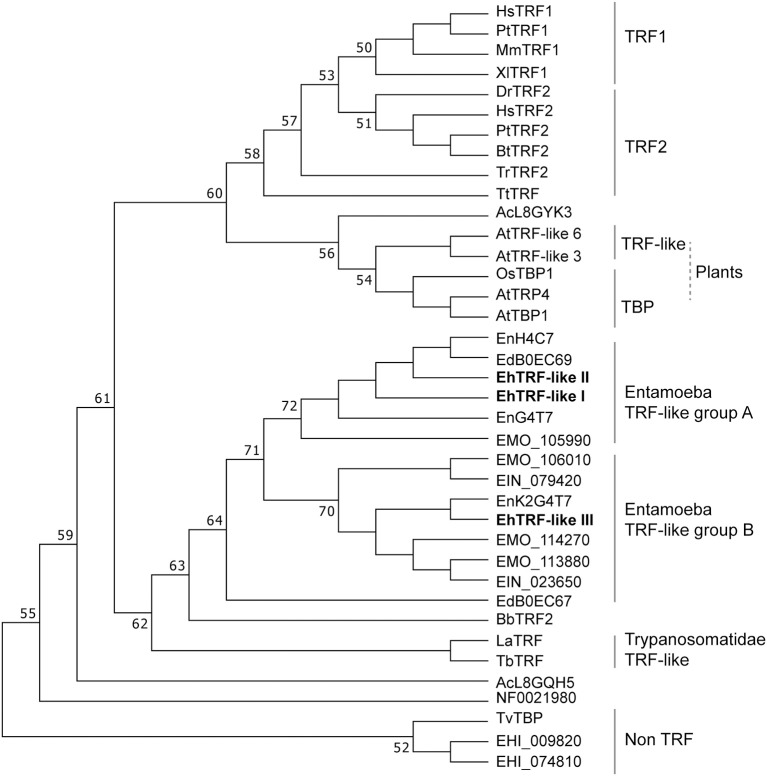
Phylogenetic tree of TRF proteins. The amino acid sequence of DBD MYB from TRF proteins was aligned using ClustalW2 and phylogenetic analysis was inferred using Neighbor-Joining method. Evolutionary distances were computed by the Poisson correction method. Bootstrap values >50% (from 1,000 replicates) are shown near to the individual branches. Evolutionary analyses were conducted using MEGA 7 (Kumar et al., 2016). HsTRF1 (Homo sapiens, NP_059523.2), MmTRF1 (*Mus musculus*, NP_033378.1), PtTRF1 (*Pan troglodytes*, XP_016815057.1), XlTRF1 (*Xenopus laevis*, DQ146827.1), HsTRF2 (*H. sapiens*, NP_005643.2), PtTRF2 (*P. troglodytes*, XP_016785565.1), BtTRF2 (*Bos taurus*, NP_001341392.1), BbTRF2 (*Branchiostoma belcheri*, XP_019634930.1), DrTRF2 (*Danio rerio*, BC096857.1), TrTRF2 (*Thecamonas trahens*, XP_013759355.1), TtTRF (*Takifugu rubripes*, XP_011616883.1), AtTRF-like 3 (*A. thaliana*, NP_001185023.1), AtTRF-like 6 (*A. thaliana*, NP_974133.1), AtTBP1 (*A. thaliana*, XP_015625433.1), AtTRP4 (*A. thaliana*, NP_196886.1), OsTBP1 (*Oryza sativa*, XP_015625433.1), EhTRF-like I (*E. histolytica*, XP_649878.1), EhTRF-like II (*E. histolytica*, XP_649880.1), EhTRF-like III (*E. histolytica*, XP_657528.1), EdBOEC67 (*Entamoeba dispar*, XP_001735901.1), EdB0EC69 (*E. dispar*, XP_001735903.1), Ein079420 (*E. invadens*, XP_004184366.1), Ein023650 (*E. invadens*, XP_004257447.1), EnH4C7 (*E. nuttalli*, XP_008857362.1), EnK2H4C7 (*E. nuttalli*, XP_008860337.1), EnK2G4T7 (*E. nuttalli*, XP_008860339.1), NF0021980 (*Naegleria fowleri)*, LaTRF2 (*Leishmania amazonensis*, ABU53006.1), TbTRF (*Trypanosoma brucei*, AAX86992.1), TvTBP *(Trichomonas vaginalis*, XP_001314728.1), EHI_009820 (*E. histolytica*, XP_001913661.1), and EHI_074810 (*E. histolytica*, XP_649576.1).

In order to shed light into the evolutionary relationships of EhTRF-like proteins, we aligned the MYB DBD amino acid sequence of TRFs from *E. histolytica, H. sapiens*, representative vertebrates, plants and deep branching protozoa, including other members of Entamoeba genus and Trypanosomatids (Figure S3). Proteins coded by genes from *locus* EHI_009820 and EHI_074810 from *E. histolytica* and TvTBP protein from *Trichomonas vaginalis* served as outgroup for tree reconstruction. Interestingly, the alignment with the MYB DBD showed that the first tryptophan residue in TRF-like proteins from Entamoeba genus was replaced with the aromatic amino acid phenylalanine, and unlike to TRF1 and TRF2 they present serine or cysteine residues in the third α-helix of their MYB DBD. In addition, all Entamoeba TRF-like proteins conserved the Telebox signature in a greater extent than other unicellular organisms like Trypanosomatids, where homologs to TRF have been already characterized (Li et al., 2005; da Silva et al., 2010). The phylogenetic inference showed that the TRF proteins are separated into two branches, one of them included the vertebrate proteins related to TRF1 and TRF2 and the TRF-like proteins from plants, and in the other branch contained TRF-like proteins from protozoan parasites including members from the Entamoeba genus (Figure 2). It is important to highlight that all TRF-like proteins from Entamoeba genus were clustered into a unique clade and subdivided in two groups that separated members of the EhTRF-like I and II (group A) from members of the EhTRF-like III (group B). The topology observed in the phylogenetic analysis could be the result of a gene duplication event. Interestingly, genes that encoded EhTRF-like I and II proteins are contiguous located in the same genomic location (DS571146). These results suggest that TRF proteins evolved from a common ancestor before vertebrate TRFs diverged and that in the case of *E. histolytica*, a gene duplication events could occur and therefore increased the TRF gene number.

### Shelterin machinery survey

To determine whether *E. histolytica* contains other components of Shelterin machinery besides TRFs, we searched for proteins homologous to Rap1, TIN2, TPP1 and POT1 in its genome. Through this analysis, we identified a hypothetical protein (AmoebaDB AN: EHI_064550, 156 residues) with 26% identity to *H. sapiens* Rap1 (399 amino acids; Table 2). Sequence analysis of this protein revealed the presence of a TRF2-interacting telomeric protein/Rap1 C-terminal domain (*e*-value of 2e-19), suggesting that in *E. histolytica* this hypothetical protein could bind to EhTRF-like III. For these reasons, we decided to name EHI_064550 as EhRap1-like protein. In *H. sapiens*, RAP1 binds to DNA through a MYB-type domain, which was absent in EhRap1-like protein, suggesting that its function is limited to telomeres protection. By using *H. sapiens* proteins as a bait, no other members of Shelterin machinery were identified in the *E. histolytica* genome. Nevertheless, the *E. histolytica* genome encodes a protein which is annotated in AmoebaDB database as the Replication Factor A1 (AN: EHI_062980), therefore this protein was dubbed EhRpa1-like. EhRpa1-like conserves an oligonucleotide/oligosaccharide (OB) domain (e-value of 9e-50 according to Pfam), which could be used for single-stranded telomeric DNA recognition in the absence of POT1 (Table 2). These findings, support the idea that *E. histolytica* exhibits a rudimentary Shelterin-like complex conformed by EhTRF-like I, II, III, and EhRap1-like. In addition, EhRpa1-like could have a possible role in telomere maintenance.

**Table 2:** Candidate proteins to be part of the Shelterin-like complex of *E. histolytica*.

***Entamoeba histolytica***			***Homo sapiens***		
**Locus^∧^**	**Annotation^∧^**	**Amino acid size (aa)**	**% Identity**	**Score**	***E*–value**
**EhRap1-like**
EHI_064550	UP^#^	156	26%	86.7	2e-19
**EhRpa1-like**
EHI_062980	Replication factor A protein 1	310	17%	22%	9e-50

∧ *According to Amoeba data base*.

# *Putative uncharacterized protein*.

### Expression of the EHTRF-like proteins in trophozoites of *E. histolytica*

**Figure 3:**
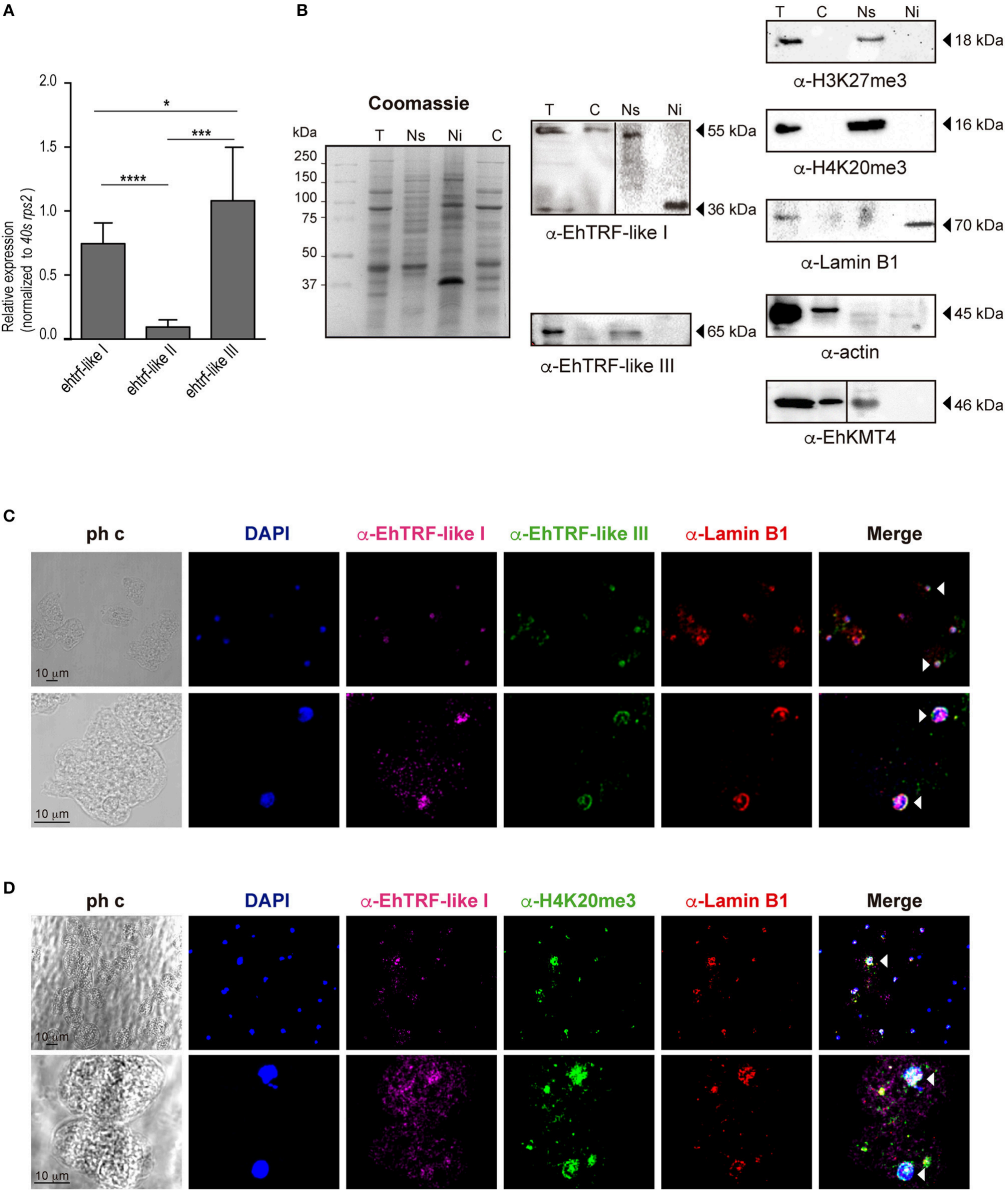
Expression of the EhTRF-like proteins in *E. histolytica* trophozoites. **(A)** The *trf-like* genes have differential expression levels. Absolute RT-qPCR quantification of *ehtrf-like* genes in trophozoites grown in basal conditions. Ribosomal *40s rsp2* subunit gene was used as a control. **(B)** Detection of EhTRF-like I and EhTRF-like III proteins in different trophozoite fractions. Coomassie-blue stained SDS-PAGE of total protein (T), nuclear soluble (Ns), nuclear insoluble (Ni), and cytoplasmic **(C)** extracts. Replicates were transferred to nitrocellulose membranes to immunodetect EhTRF-like I and EhTRF-like III with the corresponding antibodies. WB were also performed using α-H3K27me3 (as a control for Ns fraction), α-H4K20me3 (as a telomeric label in Ns fraction), α-Lamin B1 (as a control for Ni fraction), α-EhKMT4 (as control for Ns and C fractions), and α-actin (as loading control). **(C,D)** Localization of EhTRF-like proteins in *E. histolytica*. Trophozoites were processed for immunofluorescence and incubated with α-EhTRF-like I (Alexa-647, magenta), α-EhTRF-like III (Alexa-488, green), α-Lamin B1 (Alexa-555, red) and α-H4K20me3 (Alexa-488, green) antibodies. Nuclei were counterstained with DAPI and preparations were analyzed by confocal microscopy. The white arrowheads indicate colocalization in the nuclear periphery. ph c, phase contrast. *p < 0.05, ***p < 0.0002, ****p < 0.0001.

To determine whether all three *trf-like* genes were constitutively expressed by *E. histolytica*, the mRNA expression patterns were analyzed using qRT-PCR assays derived from trophozoites grown in basal culture conditions. Results showed that three *trf-like* genes had differential expression levels, being *ehtrf-like III* more apparently expressed than the other two (Figure 3A).

In order to analyse their protein expression, we produced polyclonal antibodies only against EhTRF-like I and III proteins, using synthetic peptides (N-term-CTLPSVGNALIPPS and N-term-CNKQKVQPQVSQPH, respectively) to immunize rabbits. In western blot analysis using trophozoites lysates, these antibodies revealed two bands of ~55 and 65 kDa, respectively (Figure 3B). Those bands are of higher molecular weight than the expected for EhTRF-like I (46 kDa) and III (53 kDa), respectively. Differences between theoretical and experimental molecular weights could be explained by post-translational modifications, such as phosphorylation, ubiquitination and SUMOylation of EhTRF-like I and III (Figure 1B). To determine the subcellular localization of both proteins, we carried out a cellular fractionation according to (Schreiber et al., 1989), in which cytoplasmic and soluble nuclear fractions were isolated. To extract the insoluble nuclear proteins, RIPA buffer was added to the remaining nuclei pellet. Results evidenced that EhTRF-like I was present in cytoplasmic and nuclear fractions; however, in cytoplasmic and soluble nuclear fractions the antibody recognized a 55 kDa band, while in insoluble nuclear fractions, it only detected a 36 kDa band (Figure 3B). Otherwise, EhTRF-like III protein was only observed in the soluble nuclear fraction, with the same 65 kDa molecular weight than in total lysates. In these assays, to probe the fractions purity, different cellular fractionation markers were included. As a control of the soluble nuclear fraction, we detected the K27me3 modification of H3 in the total and soluble nuclear fractions previously identified in *E. histolytica* as a repressive epigenetic mark by Foda and Singh (Foda and Singh, 2015; Figure 3B). We also detected the K20me3 modification in the H4 histone, which is specific for heterochromatin and related to telomeric regions (Blasco, 2007) and was previously identified in *E. histolytica* by Borbolla-Vázquez (Borbolla-Vázquez et al., 2015). H4K20me3 was also present in the total soluble nuclear fractions. Then, we included the detection of the enzyme methyltransferase EhKMT4 as a control of cytoplasmic and soluble nuclear fractions. This protein was detected in total, cytoplasmic and soluble nuclear extracts (Figure 3B). For the insoluble nuclear fraction, we used Lamin B1, which has been reported at the nuclear periphery in contact with the inner side of nuclear envelope in this parasite (Lozano-Amado et al., 2016). This protein was only detected in the total and insoluble nuclear fractions (Figure 3B), as expected. Additionally, we used actin as loading control for all cellular fractionations; however, a lesser amount of protein was detected in insoluble and soluble nuclear fractions than in cytoplasm, maybe due to different polymerization state of the actin within the nucleus. Despite, gel stained with Coomassie blue of the fractions obtained, demonstrated a similar protein amount in all samples (Figure 3B). Our results revealed that *E. histolytica* trophozoites differentially express *ehtrf-like I, ehtrf-like II* and *ehtrf-like III* transcripts, and the EhTRF-like I and III proteins are concentrated at the nucleus.

### EhTRF-like I and III are nuclear proteins that co-localize with lamin B1 and H4k20me3

In order to confirm the EhTRFs-like proteins localization, we performed immunofluorescence assays. Trophozoites cultured in basal conditions were processed for immunofluorescence using α-EhTRF-like I, α-EhTRF-like III, α-Lamin B1 and α-H4K20me3 antibodies coupled to Alexa-647,−488,−555 and−488, respectively. Confocal images evidenced EhTRF-like I and III mainly at trophozoite nuclei and EhTRF-like I was also localized at cytoplasm (Figure 3C). Staining of both proteins appeared at nuclear periphery, thus we employed Lamin B1 as a specific marker of this localization (Goldman et al., 2002; Lozano-Amado et al., 2016). Images revealed that EhTRF-like I and III co-localized with Lamin B1 at nuclear periphery, but EhTRF-like I presented a more diffused stain pattern inside nuclei. Therefore, we also investigated if EhTRF-like I was present in telomeric regions, employing H4K20me3 as a telomeric chromatin marker (Blasco, 2007). We found that EhTRF-like I colocalized with H4K20me3, showing sometimes diffused patterns or well-defined *foci* (Figure 3D). These results indicated that EhTRF-like I and III proteins are localized in specific nuclear regions as nuclear periphery or distributed in *foci*, co-localizing with Lamin B1 and H4K20me3. Considering the localization of EhTRF-like proteins they could be participating in the protection of the chromosome terminal ends of *E. histolytica*.

**Figure 4:**
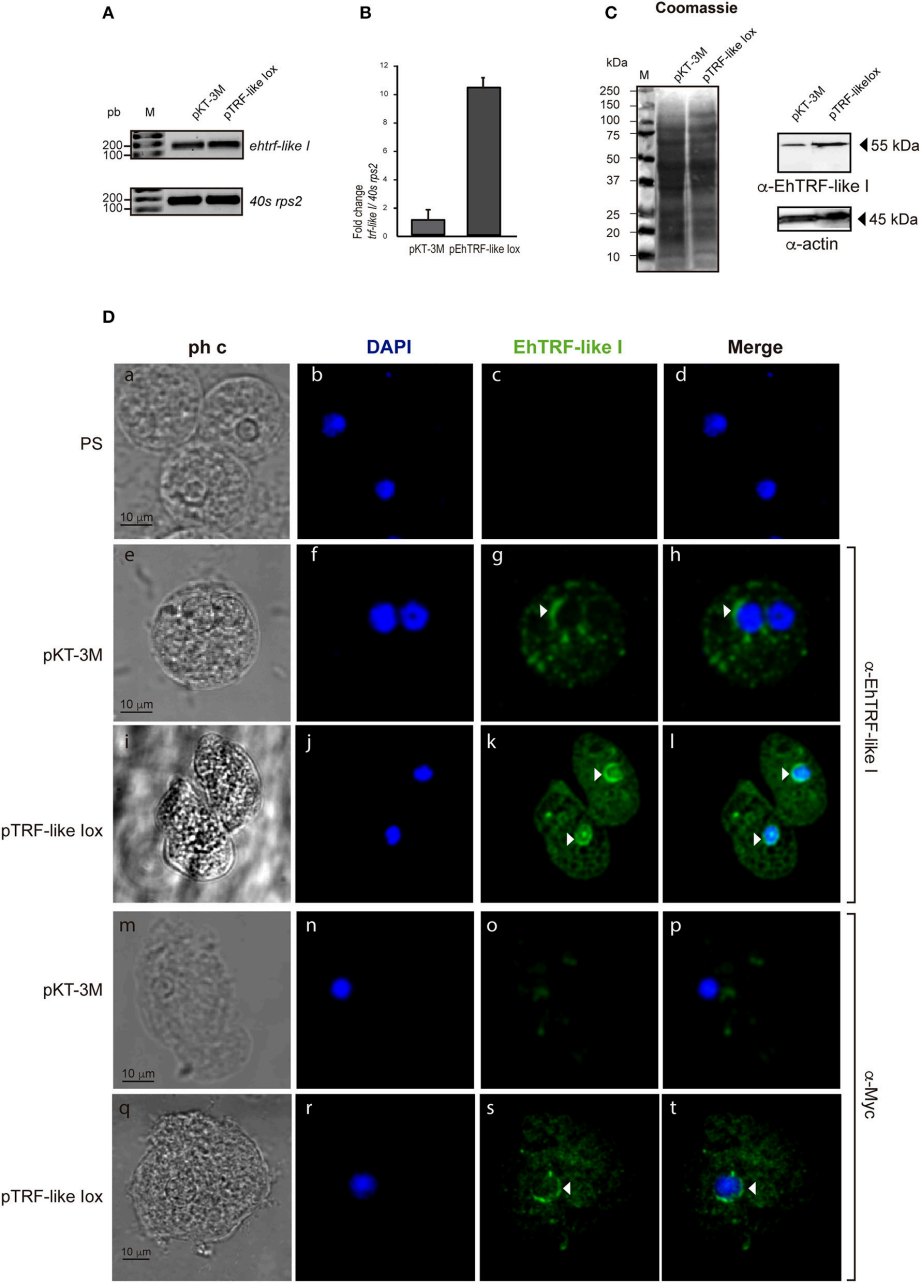
Overexpression of EhTRF-like I in *E. histolytica* trophozoites. **(A)** RT-PCR analysis of the *ehtrf-like I* gene in stably transfected trophozoites with empty plasmid *pKT-3M* or *pEhTRF-like Iox*. Ribosomal *40s rsp2* subunit gene was used as control. **(B)** Relative expression of *ehtrf-like I* gene was determined by qRT-PCR, using the *40s rsp2* gene as control. **(C)** Coomassie blue stained SDS-PAGE showing total extracts of transfected trophozoites (empty plasmid *pKT-3M* or *pEhTRF-like Iox*). A duplicate gel was transferred to nitrocellulose membrane and submitted to WB using α-EhTRF-like I and α-actin antibodies. Anti-Actin antibody was used as loading control. **(D)** Transfected trophozoites were processed for immunofluorescence and incubated with pre-immune serum (PS), α-EhTRF-like I or α-Myc antibodies, followed by the α-rabbit FITC-coupled secondary antibody. Nuclei were stained with DAPI and preparations were visualized by confocal microscopy. Arrowheads: EhTRF-like I location at nuclei. ph c, phase contrast.

**Figure 5:**
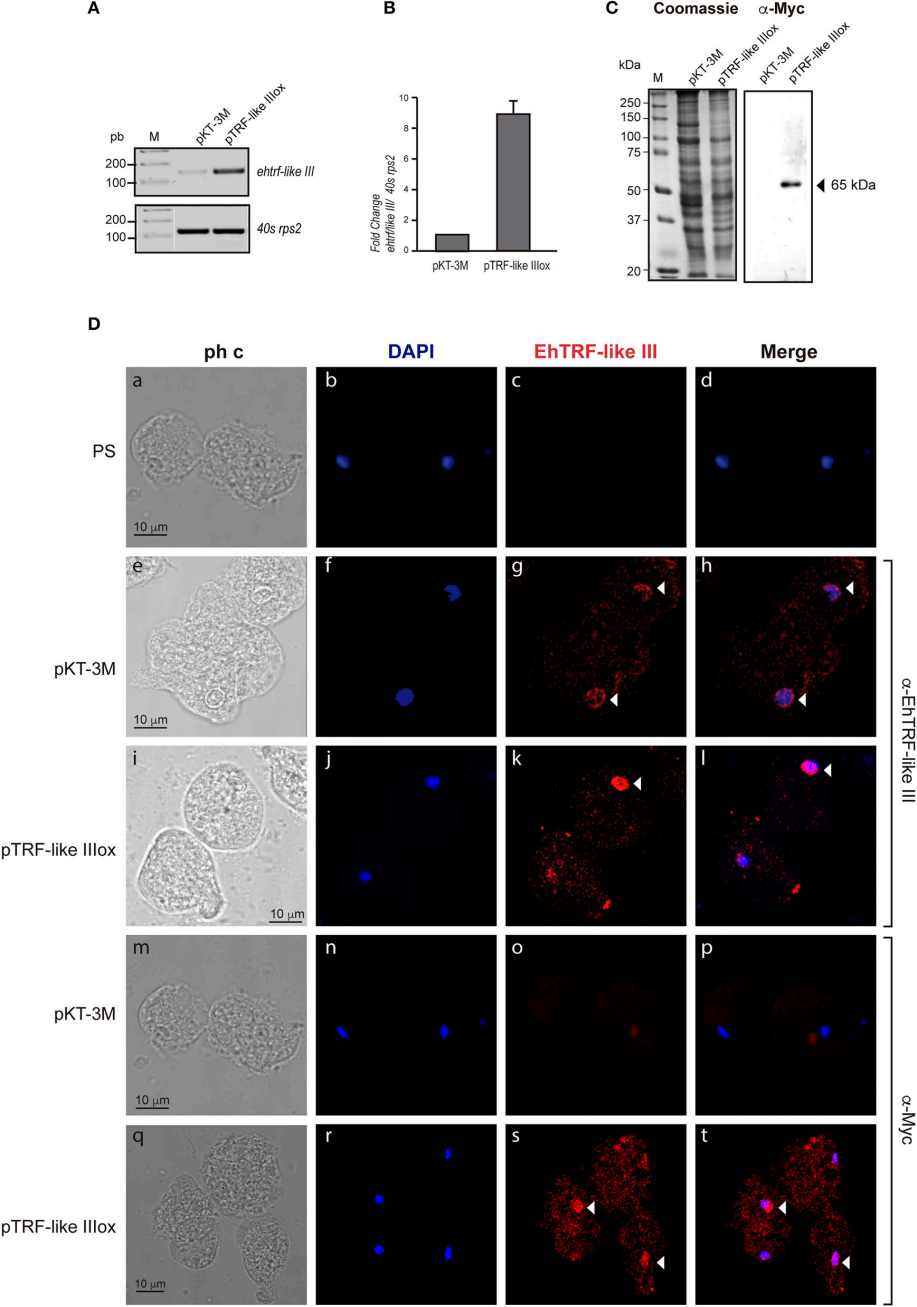
Overexpression of EhTRF-like III in *E. histolytica* trophozoites. **(A)** RT-PCR analysis of the *ehtrf-like III* gene in stably transfected trophozoites with empty plasmid *pKT-3M* or *pEhTRF-like IIIox*. Ribosomal *40s rsp2* subunit gene was used as control. **(B)** Relative expression of *ehtrf-like III* gene was determined using qRT-PCR and the *40s rsp2* gene as control. **(C)** Coomassie-blue stained SDS-PAGE showing total extracts of transfected trophozoites (empty plasmid *pKT-3M* or *pEhTRF-like IIIox*). A duplicate gel was transferred to nitrocellulose membrane and submitted to WB using α-Myc antibody. **(D)** Transfected trophozoites were processed for immunofluorescence and incubated with pre-immune serum (PS), α-EhTRF-like III or α-Myc antibodies, followed by the α-rabbit TRITC-coupled secondary antibody. Nuclei were stained with DAPI and preparations were visualized by confocal microscopy. Arrowheads: EhTRF-like III location at nuclei. ph c, phase contrast.

To validate the localization and to gain insight into the functional effect of EhTRF-like proteins, in *E. histolytica* trophozoites, we over-expressed the *ehtrf-like I and III* genes fused to the Myc tag and cloned in the *pKT-3M* vector to generate the *pTRF-like Iox and pTRF-like IIIox* overexpression vectors. Trophozoites were transfected with the *pTRF-like Iox, pTRF-like IIIox* or empty (*pKT-3M*) plasmid and stably selected in medium supplemented with 20 μg ml^−1^ G-418. According to semi-quantitative and quantitative RT-PCR results, the expression of *ehtrf-like I and ehtrf-like III* was 10.2 and 8-fold higher, respectively compared to the expression in *pKT-3M* transfected trophozoites (Figures 4A,B, 5A,B). In agreement, WB experiments of lysates from *pEhTRF-like Iox* showed that EhTRF-like I was overexpressed (55 kDa band corresponding to EhTRF-like I), when they are compared with lysates derived from trophozoites transfected with the empty vector (Figure 4B). Similarly, WB experiments of lysates from *pEhTRF-like IIIox* transfected trophozoites and using α-Myc antibody, detected a 65 kDa band corresponding to EhTRF-like III (Figure 5C). As expected, in *pKT-3M* transfected trophozoites this antibody did not recognize any protein. EhTRF-like I and EhTRF-like III overexpression was confirmed in confocal images of *pEhTRF-like Iox* and *pEhTRF-like IIIox* transfected trophozoites and using the α-EhTRF-like I or α-EhTRF-like III antibody. In these parasites, proteins were more abundant at the nucleus, but they were also observed at cytoplasm (Figures 4D, 5D,j–l), in comparison to the lesser staining of trophozoites transfected with empty vector (Figures 4D, 5D,f–h). Similar nuclear and perinuclear staining were obtained using the α-Myc antibody, which detected only the heterologous proteins in trophozoites overexpressing EhTRF-like I and EhTRF-like III (Figures 4D, 5D,r–t). Comparison with trophozoites transfected with the empty vector no staining was detected (Figures 4D, 5D,n–p). No signal was obtained in trophozoites incubated with both pre-immune serums (Figures 4, 5,b–d). All of these data confirm the nuclear localization pattern of the EhTRF-like I and III proteins.

### EhTRF-like III localizes at nuclear heterochromatin regions

**Figure 6:**
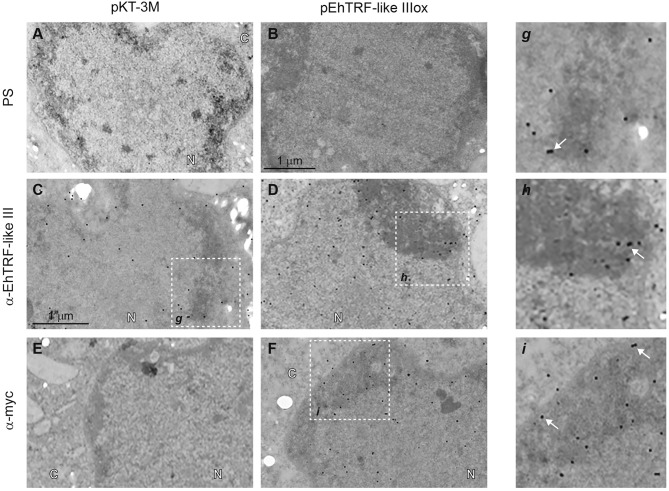
Detection by TEM of EhTRF-like III at *E. histolytica* trophozoites nuclei. TEM of transfected trophozoites (*pKT-3M* and *pEhTRF-like IIIox*) incubated with pre-immune serum (PS; **A,B**), α-EhTRF-like III **(C,D)**, or α-Myc antibodies **(E,F)**, followed by the incubation with α-rabbit gold-labeled secondary antibody. Panels g–i: magnifications of squares on **(C,D,F)**, respectively. N, nuclei and C, cytoplasm. Arrows: gold particles.

Results of EhTRF-like III localization at nuclear *foci* (Figures 3–5), suggested this protein could act in specific and functional regions of the nuclei. Thus, we analyzed the localization of this protein by transmission electron microscopy (TEM) in *pEhTRF-like IIIox* transfected trophozoites. We found EhTRF-like III abundantly in the trophozoite nuclei, enriched in the heterochromatin or highly condensed chromatin regions, close to the nuclear periphery (Figures 6C,D,g,h), using the α-EhTRF-like III antibody. This location was corroborated using the α-Myc antibody (Figure 6F,i). No signal was obtained in trophozoites incubated with pre-immune serum (Figures 6A,B) or in *pKT-3M* transfected trophozoites stained with the anti-Myc antibody (Figure 6E). These data showed that EhTRF-like III is found in chromatin regions with high degree of compaction, which is suggestive of telomeric areas, where EhTRF-like proteins could protect the chromosomes terminal ends.

### EhTRF-like III forms DNA-Protein complexes with telomeres related sequences

**Figure 7:**
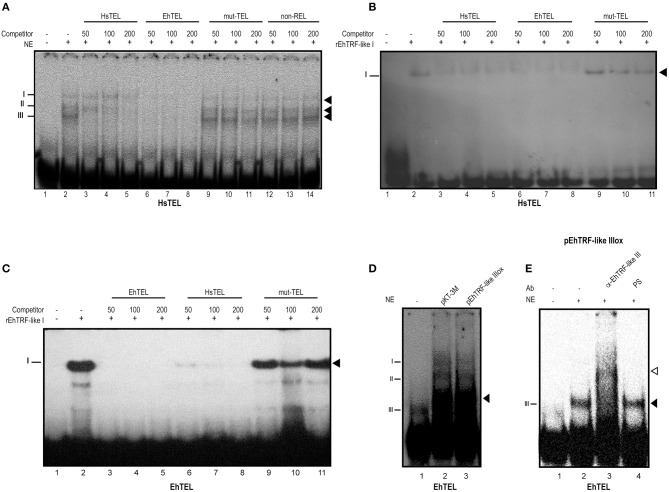
TRF-like III forms DNA-protein complexes with telomeric sequences. **(A)** EMSA was done using radiolabeled double-stranded human canonical telomeric DNA (HsTEL) as probe and nuclear extracts obtained from *E. histolytica* trophozoites. Competition assays were done in the presence of 50, 100, and 200-fold excess of non-labeled sequences: HsTEL specific competitor, *E. histolytica* sequence (EhTEL), mutated telomeric sequence (mut-TEL) or non-related sequence (non-Rel). **(B)** EMSA assay using rEHTRF-like I recombinant protein and HsTEL probe competition assays were done in the presence of 50, 100, and 200-fold excess of non-labeled sequences (HsTEL, EhTEL and mut-TEL). **(C)** EMSA assay using rEHTRF-like I and EhTEL competition assays were done in the presence of 50, 100, and 200-fold excess of non-labeled sequences (HsTEL, EhTEL and mut-TEL). **(D)** EMSA assay using EhTEL sequence and nuclear extracts obtained from trophozoites transfected with the *pKT-3M* or *pEhTRF-like IIIox* plasmids. **(E)** Super-shift assay was done using radiolabeled double-stranded EhTEL sequence as probe, nuclear extracts obtained from transfected trophozoites and α-EhTRF-like III antibody or pre-immune serum (PS). Protein-DNA complexes were separated in a 6% PAGE. Black arrowheads: specific protein-DNA complexes. Open arrowhead: super shifted protein-DNA complex.

TRF proteins bind as homodimers to double-stranded DNA telomeric sequences (Broccoli et al., 1997b; Blasco, 2007). Hence, we analyzed if in the nuclear extracts obtained from this parasite there were proteins that could recognize telomeric canonical sequences. We employed EMSA assays using nuclear extracts obtained from wild type trophozoites and the human telomeric sequence (HsTRF). The nuclear extracts from wild-type trophozoites formed three DNA-protein complexes (Figure 7A, lane 2). The presence of these three complexes is possibly due by the TRF homologs binding to the HsTEL DNA. Presumably, the formation of these complexes are due to the binding of the three EhTRF-like proteins with the HsTEL probe. In the absence of nuclear extract, no DNA-proteins complexes were formed (Figure 7A, lane 1). The HsTEL probe competed with the formation of the three complexes when it was added at increased concentrations (50, 100 and 200 molar). These results suggested that proteins forming these complexes are related to telomeric sequences (Figure 7A, lanes 3–5). Even more, when the competition was performed with an *E. histolytica* sequence, the EhTRF probe, it was more efficient (Figure 7A, lanes 6–8), showing a greater affinity for this sequence. Other mutated or non-related probes as mut-Tel or non-REL, did not competed with any of DNA-protein complexes (Figure 7A, lanes 9–14).

To investigate whether EhTRF-like proteins were able to bind to double-stranded telomeric DNA, we purified the EhTRF-like I recombinant protein (rEhTRF-like I) (Figure S4). As shown in Figures 7B,C, rEhTRF-like I bound specifically to the HsTEL and EhTEL probes. Competition assays showed that the formed complex by the recombinant protein was abolished in the presence of unlabeled HsTEL and EhTEL excess probes (Figures 7B,C, lanes 3–8). There was no competition for binding when the mut-TEL probe was used with the same molar excess (Figures 7B,C, lanes 9–11).

Next, we used the EhTEL probe and nuclear extracts derived from transfected trophozoites. In these EMSA assays, we also obtained three DNA-protein complexes, but the complex III was enriched when nuclear extracts from *pEhTRF-like IIIox* transfected trophozoites were used (Figure 7D, lane 3). To corroborate the identity of the proteins that formed the DNA-protein complex, a super-shift assay was performed using the oligonucleotide EhTEL, nuclear extracts from *pEhTRF-like IIIox* transfected trophozoites and the α-EhTRF-like III antibody. We observed a shifted complex in the presence of the α-EhTRF-like III antibody (Figure 7E, lane 3) and the pre-immune serum did not modify the formation of any DNA-protein complexes (Figure 7E, lane 4). We also included an anti-Myc antibody but in our conditions, we didn't obtain slower migration complexes, probably the Myc epitope become hidden upon binding to DNA (inducing a conformational change). This data indicated that pTRF-like III is able to form DNA-protein complexes with EhTEL sequences. Overall, these results indicated that in the nucleus of this parasite there are proteins interacting with telomeric sequences.

## Discussion

All eukaryotes protect their chromosome ends through telomere binding proteins, which are well-conserved among these organisms (Linger and Price, 2009). These proteins conform a protein complex dubbed Shelterin and bind specifically to telomeric regions in mammalian organisms (de Lange, 2005). However, less complex machineries are present in fission yeast, protozoan ciliates, and plants (Watson and Riha, 2010). The stable interaction of Shelterin with telomeres depends on the association of two proteins, TRF1 and TRF2 to double-stranded telomeric repeats. The presence of TRF-like proteins in primitive unicellular eukaryotes is outstanding and might represent the ancestral scenario during evolution of telomeres and its protein counterparts.

*In silico* analysis showed that *Entamoeba histolytica* has three genes coding for TRF-like proteins. These proteins conserve the Telobox motif in their MYB DBD, which is highly conserved as in higher eukaryotes, and showed high identity (25 to 35%) with the amino acid sequences of the human Sheltering proteins TRF1 and TRF2. It is very interesting that this parasite has three genes that encode for TRF proteins, which could be the result of a gene duplication event. It has been proposed that the selective pressure through mechanisms of recombination were involved during TRF paralog formation. Stress responses is a selection pressure which generate elevated paralog formation and lead to an exceedingly high rate of Telomeric Binding Proteins evolution (Lustig, 2016). In the case of *E. histolytica*, the host's environment submits the parasite to a variety of stress conditions (oxidative stress derived from the immune response, tissue invasion, migration, or the simply need of persistence in the host). It has been proposed that gene duplication is the main process by which new genetic material is obtained by an organism. Our qRT-PCR results showed a differential expression pattern of *Ehtrf-like* genes. *ehtrf-like III* is more expressed in basal conditions while *ehtrf-like II* has the minor expression. These results suggest that the mechanisms that control gene expression could have changed depending on the environment conditions. This differential behavior of the trophozoite has been observed changing the growing conditions of the parasite (Weber et al., 2016). The presence of three e*htrf-like* genes may result in a differential functionality of telomeric binding proteins improving the organisms' responses to the environmental challenges and protecting their chromosome ends.

Few telomeric binding proteins have been identified in protozoan parasites. Our analyses suggested that *E. histolytica* has a simpler machinery to protect their telomeric DNA. This machinery Shelterin-like could be similar to other unicellular parasites, such as *Trypanosoma* and *Leishmania*, were orthologs of TRF-2, Rpa-1 (replication protein A subunit 1) and RAP1 have been identified and characterized, suggesting that the telomeric machinery evolved early in eukaryotes (Lira et al., 2007; Yang et al., 2009).

EhTRF-like I and III proteins span from 404 to 460 amino acids with theoretical molecular weight (MW) of 46.8 to 53.1 kDa, respectively; However, in our experiments, EhTRF-like I was recognized at a 55 or a 36 kDa band and EhTRFlike III was observed in all fractions at 60 kDa band. The MW increase observed in both proteins could be related to post-translational modifications (PTMs), such as phosphorylation, ubiquitinations and SUMOylations that change protein mobility (Audagnotto and Dal Peraro, 2017). This is relevant since the function of the TRF proteins, their ability to bind to the telomeric DNA, their dimerization and location as well as their degradation and interaction with other proteins (Walker and Zhu, 2012) is regulated through PTMs. Therefore, we performed an *in silico* analysis and found that EhTRF-like proteins can be modified by SUMOylations in different residues, some of them conserved with respect to TRF-1 and TRF-2. The K302 and K303 SUMOylation sites of EhTRF-like I were conserved with respect to TRF1 (K338 and K339), which explain the MW difference since it has been reported that SUMOylated proteins increase their MW from 8 to 17 kDa for each unit of the bound SUMO peptide (Hilgarth and Sarge, 2005). We propose similar scenario for EhTRF-like III. SUMOylation regulates proteins with nuclear function since it is related to nuclear transport, transcription, location in subnuclear compartments, chromatin organization, DNA damage repair of DNA and is linked to cell cycle regulation, growth and apoptosis (Flotho and Melchior, 2013). In TRF1, SUMOylation is related to telomere maintenance through the ALT pathway (Alternative Lengthening of Telomeres), which occurs in the absence of telomerase. SUMOylated TRF-1 is recruited to the PML bodies where telomeric regions have been identified DNA (Yu et al., 2007; Royle et al., 2008). Therefore, the increase in the MW of the EhTRF-like I and III proteins could be explained by means of this PTM that suggest a similar role. Interestingly, all the enzymes involved in SUMOylation have been identified in *E. histolytica* (Bosch and Siderovski, 2013). This allows us to propose that EhTRF-like I could have similar PTMs as TRF-1 to modulate its activity and protect telomeric DNA. Detection of the EhTRF-like I protein in the insoluble nuclear fraction with a molecular weight of 36 kDa was also obtained. This molecular weight is lower than the predicted for EhTRF-like I. EhTRF-like I protein contains different residues susceptible to proteolytic cleavage, E352 was predicted as proteolytic cleavage site. If this site is functional, it would generate a peptide with a similar weight to that we found in the insoluble fraction. Interestingly, this residue is also conserved in *H. sapiens* TRF1. Finally, it has been reported that the presence of high content of acidic residues might affect the gel mobility shift of a protein and thereby explain the molecular weight variations found (Guan et al., 2015).

Human cells telomeres are tethered to the nuclear envelope during post-mitotic nuclear assembly. TRF proteins are associated with the nuclear membrane through Lamin B1. Binding of lamins to telomeres is partially mediated by TRF2, via its interaction with RAP1 which interacts with the nuclear envelope protein Sun1 (Hediger et al., 2002; Crabbe et al., 2012; Gonzalo and Eissenberg, 2016). In agreement, we found that EhTRF-like I and III colocalized with Lamin B1 at the nuclear periphery suggesting that they occupy a similar position. In accordance with this localization, in EhTRF-like III over-expressing trophozoites, this protein was localized also in the nuclear periphery proximal to the nuclear membrane in condensed heterochromatin regions. Likewise, telomeres have been found located in interphase nuclei close to the nuclear envelope; However, not in all organisms telomeres occupy this position, for example in plants like *A. thaliana* they have been reported close to the nucleolus (Schrumpfová et al., 2014). Therefore, the subnuclear location of telomeres is species-, cell type- and cell-phase dependent (Giraud-Panis et al., 2013). Telomeres carry features of repressive chromatin associated with constitutive heterochromatin. Different histone signatures have recently been identified associated with mammalian telomeres: trimethylation of H3K9 and H4K20 (Blasco, 2007). Here we selected H4K20me3 because it was previously identified and described in this parasite (Borbolla-Vázquez et al., 2015) and could suggest its participation in the organization and regulation of telomeric DNA in the *E. histolytica* nuclear periphery. Consistent with this, we observed that EhTRF-like I colocalized with the H4K20me3 mark suggesting that EhTRF-like proteins occupy regions of silenced compacted chromatin, in consistence with the telomere compacted structure (Giraud-Panis et al., 2013). Given the position of EhTRF-like I and III at the nuclear periphery and their colocalization with Lamin B and the trimethylated H4K20, we propose that these proteins could participate in the protection of chromosome ends.

Finally, we explored whether nuclear proteins from *E. histolytica* recognized the human telomeric sequence (HuTRF). Interestingly, we found three DNA-protein specific complexes from nuclear extracts of trophozoites that were competed with the human canonical telomeric sequence and with the probe derived from the STR repeat of the NK2 array that could be related to telomeric DNA (EhTEL). These DNA-protein complexes were not competed with mutated telomeric sequence (mut-TEL) or non-related (non- REL), showing specificity for telomeric sequences. Moreover, in an attempt to evaluate the telomeric DNA binding properties of EhTRF-like proteins, we observed that rEhTRF-like I recognized both HuTRF and EhTEL sequences. Since *E. histolytica* has three genes that code for three proteins with telomeric MYB DBD it would be interesting to determine if DNA-protein complexes correspond to these three proteins. Using nuclear extracts from trophozoites overexpressing EhTRF-like III we found that this protein formed DNA protein complexes with the STR sequence of *E. histolytica*. In our conditions, only complex III was enriched when extracts from TRF-like III overexpressing clones were used with the EhTEL probe indicating that the TRF-like III protein interacts with the STR repeat of the tRNA genes. This is the first report were a sequence derived from the tRNA arrays ([NK2]) is used to determine its recognition by nuclear proteins and rTRF-like I in EMSA assays. Previously, the YE array was used in fluorescence *in situ* hybridization (FISH) analysis, detecting six distinct signals at the parasite nucleus (Willhoeft and Tannich, 2000). However, as the ploidy of *E. histolytica* remains to be determined the interpretation of this evidence was difficult. It will be interest to determine the *in vivo* association of EhTRF-like proteins with sequences derived from the tRNA arrays.

**Figure 8:**
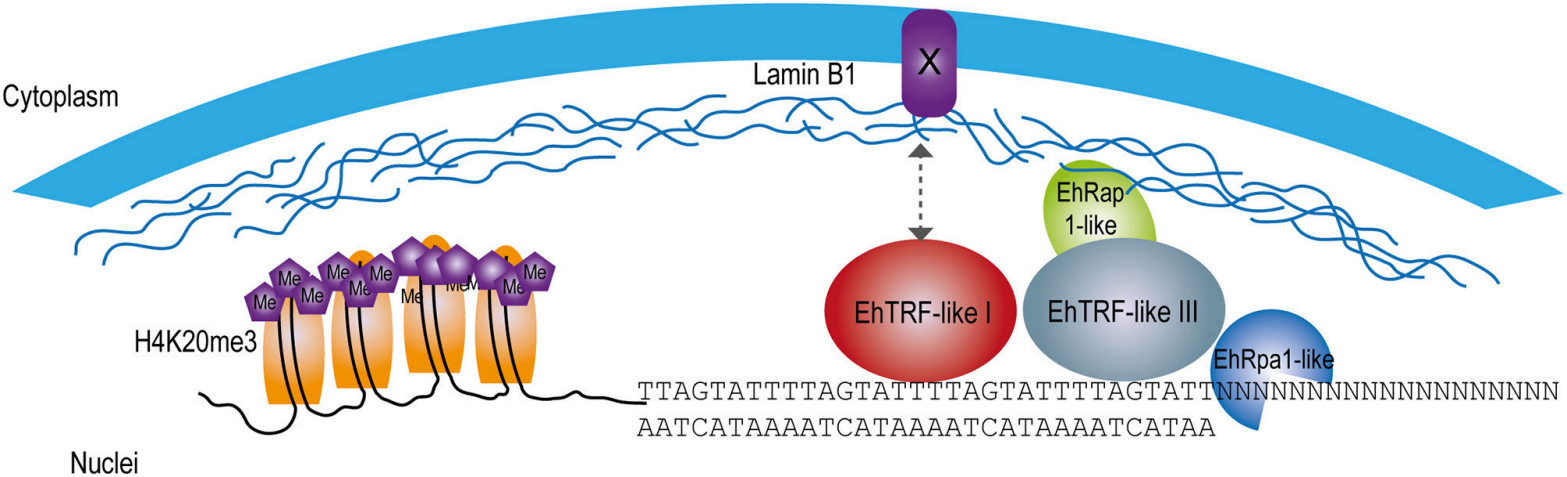
Model of EhTRF-like participation in the protection of chromosome ends in *E. histolytica*. Scheme at the nuclear periphery location of EhTRF-like I and EhTRF-like III linked to the double-stranded DNA. These proteins are close to nuclear lamina, co-localizing with the telomeric heterochromatin label H4K20me3. EhTRF-like I and III could interact with lamin B1, probably through an unknown protein with a similar function to the Sun anchoring protein as in *H. sapiens* (Giraud-Panis et al., 2013). Additionally, some putative Shelterin proteins are shown: EhRap1-like (EHI_064550) and the single-stranded DNA-binding protein EhRpa1-like (EHI_062980).

In conclusion, the protection of the chromosome ends is critical to the survival of any cell as their disruption can induce genomic instability and consequently compromise the viability of the organism. Although no canonical Shelterin proteins have been identified in this protozoan, TRF-like proteins might accomplish this role for their ability to recognize and interact with telomeric DNA through their MYB-DBD. Therefore, this work shows the first evidence of telomeric proteins in *E. histolytica* able to interact with the proposed *Entamoeba* telomeric DNA sequences conforming a simple Shelterin complex as in other protozoans (Figure 8). This work raises several interesting questions and further investigation will contribute to a better understanding of the role of EhTRF-like proteins in the telomeric function in *E. histolytica*.

## Author contributions

All authors contributed equally to design and conception of this work. FR-G, VÁ-H, EC-O, and RC-G collected *E. histolytica* experimental data. HC-H performed the *in-silico* analysis; BC-M and AL-G performed MET analysis. AB contributes with confocal microscopy. FR-G, AB, JV, EO, LL-C, and EA-L contributed to experimental design, intellectual input, interpreting data and in writing the manuscript.

### Conflict of interest statement

The authors declare that the research was conducted in the absence of any commercial or financial relationships that could be construed as a potential conflict of interest.
