# Supplementary material for: Telomeric Repeat-Binding Factor Homologs in Entamoeba histolytica: New Clues for Telomeric Research
Source: Front Cell Infect Microbiol. 2018 Oct 2;8:341. doi: 10.3389/fcimb.2018.00341 (PMC6175992; doi:10.3389/fcimb.2018.00341)
Supplement: Supplementary file 1 [file Data_Sheet_1.pdf]

Supplementary Material

Telomeric repeat-binding factor homologues in *Entamoeba histolytica*: new clues for telomeric sequences search

Francisco Javier Rendón-Gandarilla[1,+], Víctor Álvarez-Hernández[1], Elizabeth J. Castañeda-Ortiz[1], Helios Cárdenas-Hernández[1], Rosa Elena Cárdenas-Guerra[1], Jesús Valdés[2], Abigail Betanzos[3,4], Bibiana Chávez-Munguía[4], Anel Lagunes-Guillen[4], Esther Orozco[4], Lilia López-Canovas[1] and Elisa Azuara-Liceaga[1*].

*** Correspondence:** Corresponding Author: elisa.azuara@uacm.edu.mx

Supplementary Data

1 Supplementary Tables

Table S1. Paired primers used for assessing the transcript levels of *ehtrf-like* genes in *E. histolytica*

**Gene**	**Accession number**	**Sequences of paired primers (5´- 3´)**	**Amplicon size (pb)**
Ehtrf-1	EHI_001090	F: ACCCTCAGTTTGCAACCCTT<br>R: AGGACTTTGTTCCATTTTTACTTC	235
Ehtrf-2	EHI_001110	F: AAAAGAGCATTAGCAACGGTTAT<br>R: TTTCTGCATTTGGATCTGAACAA	240
Ehtrf-3	EHI_148140	F: GCGCGTTCTCAAATACAACC<br>R: GTTCAGAGGCTGCAGGAAGT	145
**Internal control**			
40s rps2	EHI_020280	F: ATTCGGAAATAGAAGAGGAGG	105

subunit gene	R: ACTAATCTT CCAAGCTTGGT

Table S2. Telomeric probes designed for EMSA assays.

DNA probe	Oligonucleotides	Characteristics	Reference
HsTel	Forward<br>5'-GCTCTAGGGTTAGGGTTAGGGTTAGGGTTA-3'<br>Reverse<br>5'-AATCCCAATCCCAATCCCAATCCCATCTAC-3'	Human telomeric sequence	Hwang et al., 2001
EhTel	Forward<br>5′-GCTCTATTAGTATTTTAGTATTTTAGTATTTTAGTATT-3′<br>Reverse<br>5′-AATCATAAAATCATAAAATCATAAAATCATAACGAGATCTAC-3′	The EhTEL contains one STR sequence present in the NK2 tRNA array	Tawai et al., 2008
mut-TEL	Forward<br>5′-GCTCTAGATGTGGGTAGCTAGCTCTGACTCAGG-3′<br>Reverse<br>5′-ACCCGATCGATCGAGACTGAGTCCCGAGATCTAC-3′	Mutated telomeric sequence	Hwang et al., 2001
non-Rel	Forward<br>5´-GCTCTAGATGTCGAGTCTGTGTGTACATTACGTGTG-3´<br>Reverse<br>5´-AGCTGAGACACACATGTAAATGCACACCGAGATCTAC-3´	Non-related sequence	Hwang et al., 2001

Table S3. Identity matrix of EhTRF-like of *E. histolytica* and *H. sapiens* TRF proteins

	Full-length (% identity)					MYB DBD (% identity)				
	EhTRF-like I	EhTRF-like II	EhTRF-like III	HsTFR1	HsTFR2	EhTRF-like I	EhTRF-like II	EhTRF-like III	HsTFR1	HsTFR2
EhTRF-like I	-	35.89	33.91	13.86	14.1	-	**93.44**	**89.13**	36.66	33.33
EhTRF-like II		-	63.94	13.89	13.15		-	80.32	36.66	33.33
EhTRF-like III			-	14.35	12.6			-	36.66	31.66
HsTFR1				-	**22.8**				_	**51.66**
HsTFR2					-					-

Percent of identity based on the full-length protein or the MYB DBD sequence pairwise sequence alignment.

2. Supplementary Figures

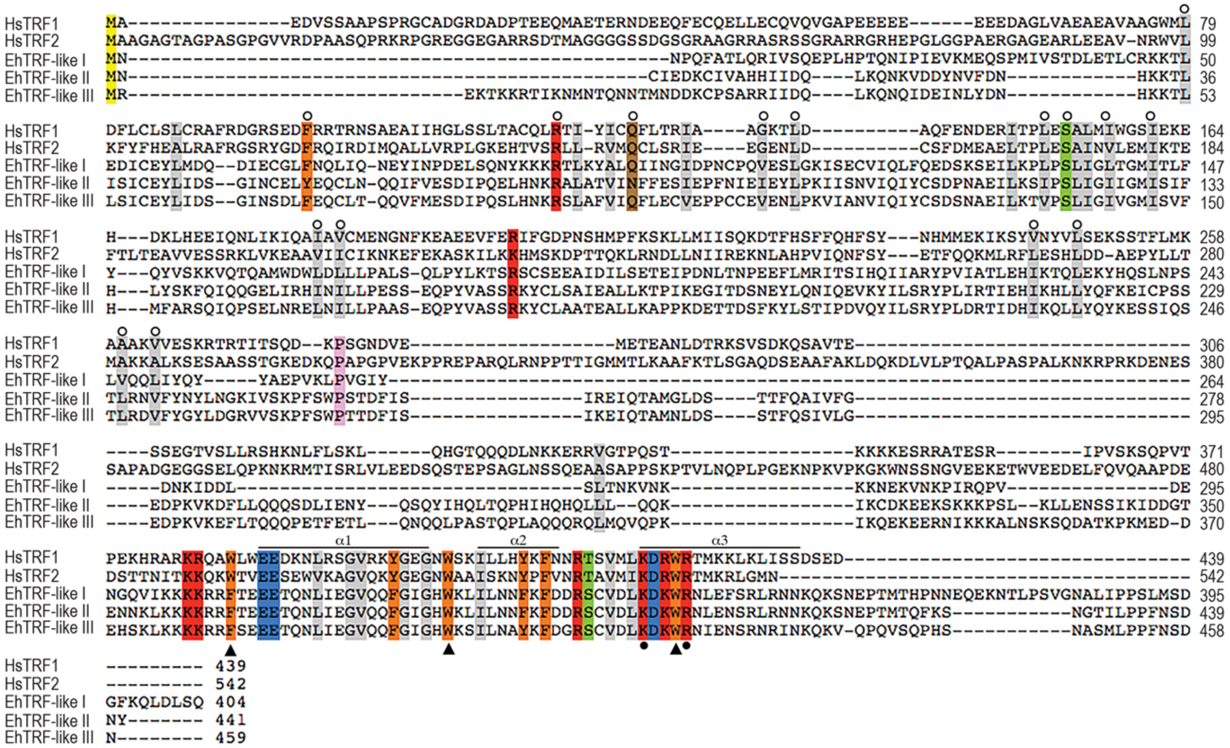

Figure S1. Alignment of complete amino acid sequences of TRF proteins from *H. sapiens* and *E. histolytica*. In the alignment, conserved hydrophobic residues involved in the dimer interphase formation such as leucines, isoleucines, or phenylalanines are shown with open circles and identical amino acids are highlighted in colors. The position of three α-helices within the MYB DBD and conserved tryptophans or aromatic residues marked with black arrowheads are also showed.

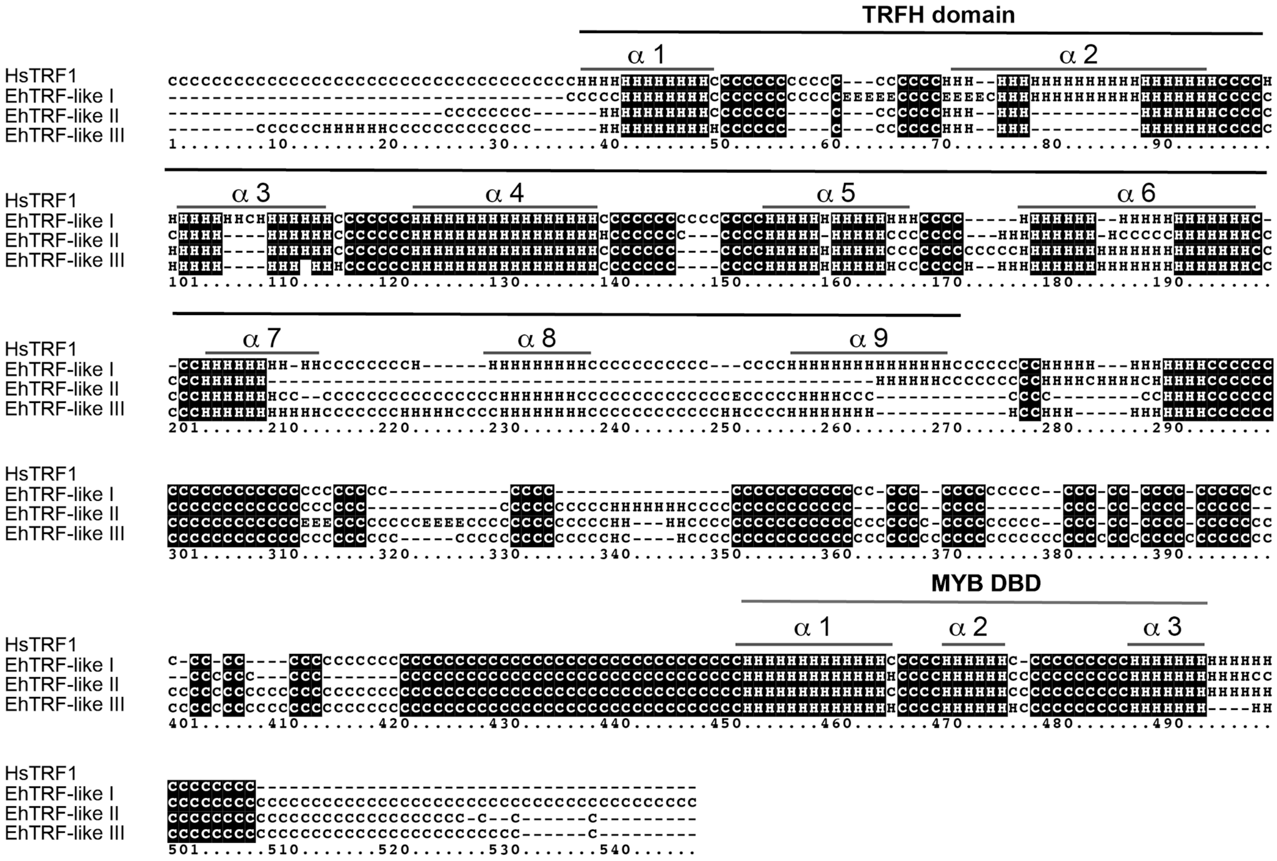

Figure S2. EhTRF-like proteins and their putative TRFH domain. Secondary structure alignment of complete sequence of HsTRF1 and EhTRF-like proteins performed in PSIPRED. E: beta sheet, H: helix and C: random coil. The nine and three α-helices of TRFH and MYB DBD domains, respectively are shown.

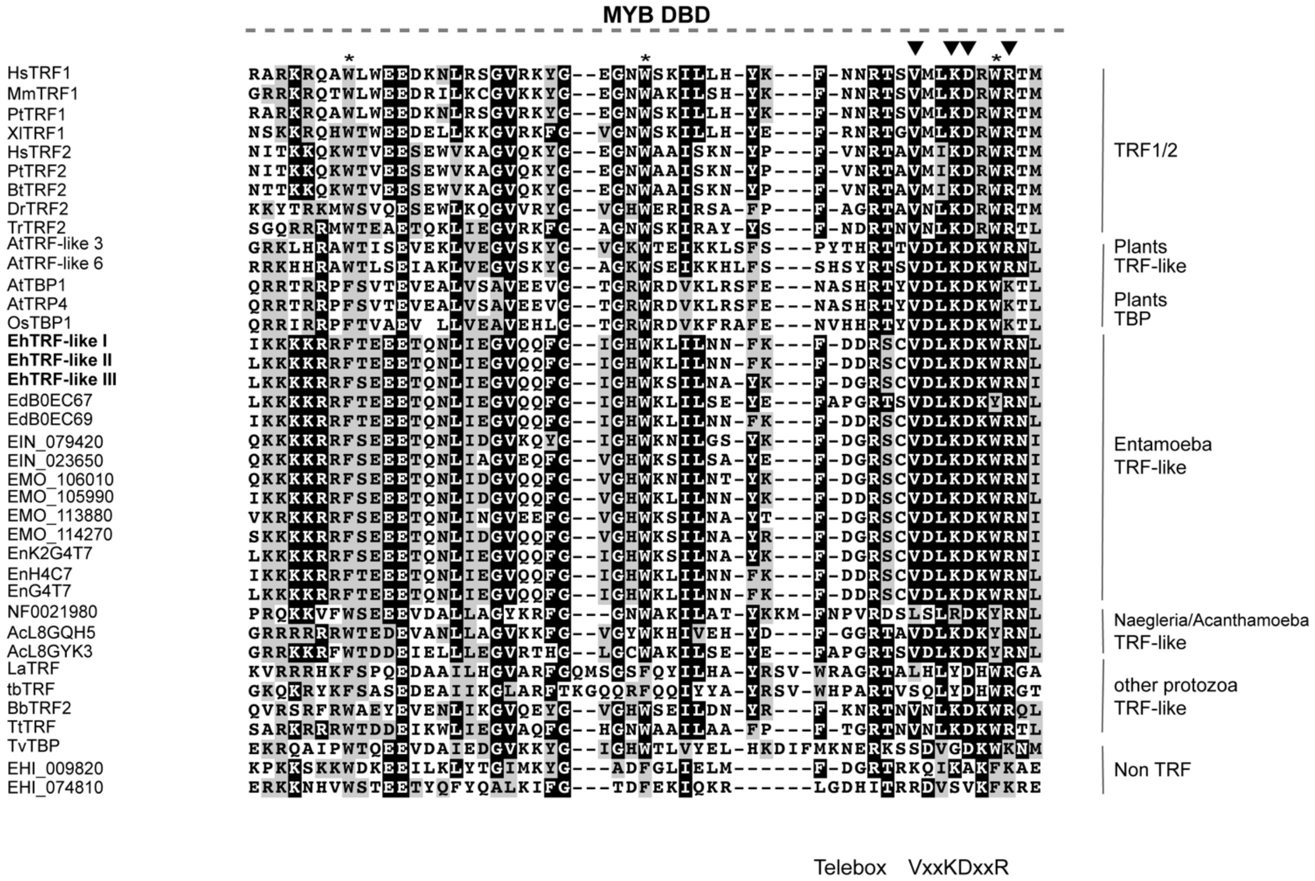

Figure S3. **Multiple alignment of the MYB DBD amino-acid sequence of several TRF proteins**. ClustalW alignment of the MYB DBD belonging to TRF proteins from different organisms. Identical amino acid residues are black boxed. Conserved tryptophans and telobox residues are showed in asterisks and arrowheads, respectively. HsTRF1 (*Homo sapiens*, NP_059523.2), MmTRF1 (*Mus musculus,* NP_033378.1), PtTRF1 (*Pan troglodytes,* XP_016815057.1), XlTRF1 (*Xenopus laevis,* DQ146827.1), HsTRF2 (*H. sapiens*, NP_005643.2), PtTRF2 (*P. troglodytes,* XP_016785565.1), BtTRF2 (*Bos taurus,* NP_001341392.1), BbTRF2 (*Branchiostoma belcheri,* XP_019634930.1), DrTRF2 (*Danio rerio,* BC096857.1), TrTRF2 (*Thecamonas trahens*, XP_013759355.1), TtTRF (*Takifugu rubripes*, XP_011616883.1), AtTRF-like-1 (*Arabidopsis thaliana*, NP_001330208.1), AtTRF-like 3 (*A. thaliana*, NP_001185023.1), AtTRF-like 6 (*A. thaliana*, NP_974133.1), AtTBP1 (*A. thaliana*, XP_015625433.1), AtTRP1 (*A. thaliana*, NP_196886.1),OsTBP1 (*Oryza sativa*, XP_015625433.1), EhTRF-like I (*E. histolytica,* XP_649878.1), EhTRF-like II (*E. histolytica,* XP_649880.1), EhTRF-like III (*E. histolytica,* XP_657528.1), EdBOEC67 (*Entamoeba dispar*, XP_001735901.1), EdB0ESZ2 (*E. dispar*, XP_001741195.1), EdB0EC69 (*E. dispar*, XP_001735903.1), Ein079420 (*E. invadens,* XP_004184366.1), Ein023650 (*E. invadens,* XP_004257447.1), EnH4C7 (*E. nuttalli,* XP_008857362.1), EnK2H4C7 (*E. nuttalli,* XP_008860337.1), EnK2G4T7(*E. nuttalli,* XP_008860339.1), NF0021980 (*Naegleria fowleri)*, LaTRF2 (*Leishmania amazonensis*, ABU53006.1), TbTRF (*Trypanosoma brucei,* AAX86992.1), TvTBP *(Trichomonas vaginalis*, XP_001314728.1), EHI_009820 (*E. histolytica*, XP_001913661.1), and EHI_074810 (*E. histolytica,* XP_649576.1).

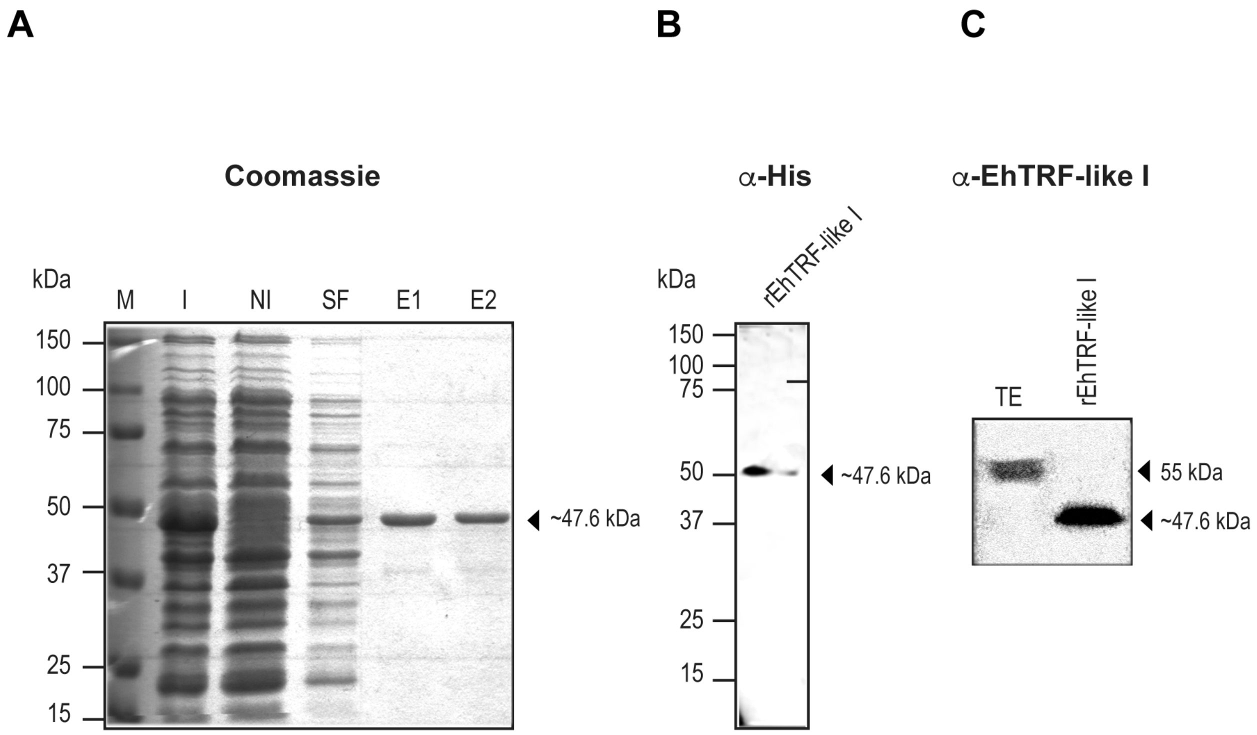

Figure S4. Expression and purification of rEhTRF-like I from *E. histolytica*. A) Coomassie brilliant blue-stained SDS-PAGE on 12% gel of recombinant EhTRF-like I. NI: Not Induced; I: Induced; SF: Soluble Fraction; E1-2: elutions from the purification by nickel affinity chromatography. B) WB assay with the anti-His antibody against the recombinant protein. C) WB assay with the anti-TRF-like I antibody in total extracts (TE) from trophozoites of *E. histolytica* or the rEhTRF-like I. The letter M and lines on the left side show the position of the molecular weight markers in kilodalton (kDa).
